# Autism Heterogeneity Related to Preterm Birth: Multi-Ancestry Results From the Simons Foundation Powering Autism Research for Knowledge Sample

**DOI:** 10.1016/j.bpsgos.2025.100614

**Published:** 2025-09-17

**Authors:** Charikleia Chatzigeorgiou, Zeynep Asgel, Marina Natividad Avila, Behrang Mahjani, Vahe Khachadourian, Tade Souaiaia, Niamh Mullins, Magdalena Janecka

**Affiliations:** a Department of Psychiatry, Icahn School of Medicine at Mount Sinai, New York, New York; b Medical Research Council Integrative Epidemiology Unit, University of Bristol, Bristol, United Kingdom; c Department of Child and Adolescent Psychiatry, New York University Grossman School of Medicine, New York, New York; d Seaver Autism Center for Research and Treatment, Icahn School of Medicine at Mount Sinai, New York, New York; e Department of Genetics and Genomic Sciences, Icahn School of Medicine at Mount Sinai, New York, New York; f Department of Artificial Intelligence and Human Health, Icahn School of Medicine at Mount Sinai, New York, New York; g Mindich Child Health and Development Institute, Icahn School of Medicine at Mount Sinai, New York, New York; h Department of Molecular Medicine and Surgery, Karolinska Institutet, Stockholm, Sweden; i Department of Medical Epidemiology and Biostatistics, Karolinska Institutet, Stockholm, Sweden; j Department of Cell Biology, SUNY Downstate, New York, New York; k Department of Genetics and Genomic Sciences, Icahn School of Medicine at Mount Sinai, New York, New York; l Charles Bronfman Institute for Personalized Medicine, Icahn School of Medicine at Mount Sinai, New York, New York; m Department of Population Health, NYU Grossman School of Medicine, New York, New York

**Keywords:** Autism, Cross-ancestry, Heterogeneity, Preterm, PRS

## Abstract

**Background:**

Autism spectrum disorder (ASD) shows significant clinical variability, likely due to a combination of genetic and environmental factors. Preterm birth is a known risk factor for ASD, occurring in approximately 13% of diagnosed individuals. While genetic factors contribute to preterm birth in the general population, the relationship between genetic variation, preterm birth, and ASD heterogeneity remains unclear.

**Methods:**

We investigated the genetic factors associated with preterm birth in 31,947 autistic individuals using data from the SPARK (Simons Foundation Powering Autism Research for Knowledge) sample. We conducted 3 ancestry-specific genome-wide association studies for African/African American, admixed American, and non-Finnish European ancestries, followed by a meta-analysis of 3308 preterm cases and 28,639 controls using METAL. Functional mapping and gene-based analyses were performed using FUMA, and genetic correlations were estimated using LDSC and Popcorn. Polygenic risk scores (PRSs) were computed with BridgePRS, using PRS of preterm birth in the general population.

**Results:**

Our study identified ancestry-specific genetic loci associated with preterm birth in ASD cases. Although the meta-analysis results were not statistically significant, the estimated single nucleotide polymorphism heritability was 14%, indicating a meaningful contribution of common genetic variants. Across ancestry groups, preterm birth status was not significantly associated with PRSs for any psychiatric or medical conditions analyzed. However, polygenic liability to preterm birth in the general population was linked to several congenital anomalies after multiple testing adjustments.

**Conclusions:**

These findings highlight the importance of diverse ancestries and early-life exposures in understanding ASD heterogeneity. Future research should replicate these findings in larger samples and explore rare variants associated with preterm birth to better understand the relationship between gestational duration and clinical and genetic differences in ASD.

## Plain Language Summary

In this study, we investigated whether autistic individuals born preterm carry different genetic risks than those born full-term. Using a large and ancestrally diverse sample, we identified genetic signals associated with preterm birth that varied by ancestry. These findings emphasize the importance of including diverse populations in genetic studies and suggest that early-life factors may help explain some of the differences seen among individuals with autism.

Approximately 1% of children worldwide (1) and 1 in 36 children in the United States (2) are diagnosed with autism spectrum disorder (ASD). ASD is characterized by differences in social communication and restricted/repetitive patterns of behaviors (3) and is often accompanied by co-occurring conditions, including attention-deficit/hyperactivity disorder (ADHD), motor delay, and anxiety disorders (4). Autism is highly heterogeneous, with variability in etiology, age of onset, and clinical symptoms and their progression, as well as the pattern of co-occurring conditions. However, both the causes of autism and the factors contributing to case heterogeneity remain largely unknown (5), and outside of research on monogenic forms of autism (6, 7, 8), few studies have mapped specific etiologic factors onto clinically meaningful subgroups.

While the causal role of early-life exposures in autism remains unclear (9), our previous research has shown that such exposures can help identify autistic subgroups with distinct co-occurring conditions (4). For example, autistic individuals born preterm were more likely to be diagnosed with difficulty gaining weight or motor delay (compared with autistic individuals born to term), while autistic individuals whose mothers reported infection during pregnancy were more likely to experience hearing loss (4). Therefore, even if exposures such as preterm birth are not causally linked to autism, they can still contribute to risk prediction (10) and can be leveraged to study autism heterogeneity. While these early-life exposures were more frequent in autistic individuals than in their unaffected siblings, their association with other co-occurring conditions was observed in both groups of children. This suggests that the factors associated with autism heterogeneity do not need to be autism specific but can be linked to similar phenotypic variation in the nonautistic population.

Given our earlier results demonstrating clinical differences among autistic individuals based on their early-life exposures (4), here we focus on one such exposure—preterm birth—to better understand the mechanisms underlying the link between early exposures and clinical variability of autism. Preterm birth—delivery before 37 weeks of gestation—is relatively common (11) and is associated with a range of child health outcomes, which worsen with earlier delivery (12). The sequelae of preterm birth extend beyond the immediate newborn phase, underscoring the imperative need for comprehensive care and support. A range of epidemiological factors are known to influence the risk of preterm birth in the general population, including maternal age, socioeconomic status, prenatal infection, and inflammation (13). Additionally, both distinct maternal and fetal genetic factors have been shown to contribute to the risk of preterm birth (14,15).

Due to the complex nature of the relationships between gestational duration and both genetic and phenotypic variation, the impact of preterm birth on autism heterogeneity may arise due to its direct (nongenetic) influence on development and children’s clinical features and/or genetic variants that pleiotropically affect risk of preterm birth and autism. Such genetic effects could occur due to both direct and indirect mechanisms (14,15), corresponding, respectively, to a child’s own genetics influencing their outcomes and maternal variants impacting the prenatal environment, with consequences for phenotypic heterogeneity.

Focusing on the genetic mechanisms, we investigated whether systematic genetic differences exist between autistic individuals born term versus preterm and the degree to which those differences can account for the phenotypic heterogeneity reported previously in autism due to genetic pleiotropy. While the genetic variants underlying preterm birth in autistic individuals and the general population likely overlap, pleiotropic loci, which influence both autism and preterm birth, may play a larger role in the former group, motivating our investigation. We used a multi-ancestry SPARK (Simons Foundation Powering Autism Research for Knowledge) cohort to detect genetic loci differentiating autistic individuals born term and preterm, and then we performed their functional analysis and examined genetic and phenotypic overlap between preterm birth and other traits. Collectively, such analyses can help shed light on the genetic landscape associated with preterm birth in autism, elucidate genetic factors contributing to case heterogeneity, and unravel the intricate relationships between preterm birth and other phenotypes in autistic individuals.

## Methods and Materials

### Sample

SPARK is a large autism study, with both online and in-person enrollment. Eligibility criteria include a diagnosis of ASD and residence in the United States. Participants are encouraged to enroll with their parents and siblings, irrespective of their ASD status. Upon enrollment, all participants and/or their caregivers complete a series of demographic, cognitive, behavioral, and medical questionnaires (16). DNA is extracted from saliva samples. Genotype data were derived from the Illumina Global Screening Array (GSA version 1, version 2, GSA_24 version 2-0_A2).

We used phenotypic and genetic data drawn from SPARK iWES version 2 data freeze. Preterm birth was defined as self- or caregiver-reported delivery before 37 weeks of gestation. Individuals with an ASD diagnosis born prematurely were defined as cases. Individuals with a diagnosis of ASD born to term were defined as controls.

### Genotyping and Imputation

#### Preimputation Quality Control

We first removed single nucleotide polymorphisms (SNPs) located within sex or mitochondrial chromosomes or with a low genotyping rate (<0.9). Subsequently, we removed samples with high genotype missingness (>0.1) and discrepancies between self-reported and genotype-derived sex. We retained SNPs with genotyping rate ≥ 0.98, minor allele frequency (MAF) ≥ 0.01, deviation from Hardy-Weinberg equilibrium *p* ≥ 10^−6^ in cases or *p* ≥ 10^−10^ in controls. Additionally, we removed related individuals, one from each pair with identity-by-descent (PI_HAT) > 0.2, retaining the individual with higher call rates within the pair.

#### Imputation and Postimputation Quality Control

Input data preparation for imputation was performed according to the data preparation guidelines provided by the TOPMed Imputation Server. Quality control (QC) for the TOPMed reference panel was carried out using the toolbox provided by Will Rayner (http://www.well.ox.ac.uk/∼wrayner/tools/). Due to the diverse ancestries of the SPARK individuals, all genotypes were imputed to a diverse reference panel (TOPMed panel; 194,512 haplotypes; version R2 on GRCh38). Genotype imputation for each chromosome was performed after the standard QC and phasing steps. Postimputation QC was performed using PLINK version 1.9 and vcftools (17) by extracting SNPs with MAF ≥ 0.01, info score ≥ 0.5, genotype missingness ≤ 1%, and Hardy-Weinberg equilibrium *p* value of ≥1 × 10^-6^.

#### Ancestry Mapping

We adopted the ancestry mapping approach developed by gnomAD (https://gnomad.broadinstitute.org/news/2023-11-genetic-ancestry/). The dataset was combined with the Human Genome Diversity Project and the 1000 Genomes Project (HGDP + 1KG) subset of gnomAD and restricted to 5000 ancestry-informative SNPs (18). Principal component analysis (PCA) was performed in the joint dataset and a random forest classifier was trained on the first 10 PCs of HGDP + 1KG samples to infer ancestry for the SPARK samples, ensuring consistency in the classification process. Only SPARK samples from admixed American (AMR), African/African American (AFR), and non-Finnish European (EUR) continental ancestry labels were retained.

### Genome-Wide Association Analysis and Meta-Analysis

A genome-wide association study (GWAS) of preterm birth was done using the imputed genotype dosage data with PLINK version 2.0 and VCF tools. PCA was performed separately for each ancestry group using linkage disequilibrium (LD)–pruned, genotyped SNPs to generate eigenvectors. We conducted 3 ancestry-specific GWASs (AFR, AMR, EUR), adjusting for the 15 ancestry-specific PCs in each (note: using 10 PCs resulted in a highly deflated genomic control factor). Then we conducted a meta-analysis of the ancestry-specific GWASs using a standard error fixed effects model with genomic control correction implemented in METAL (thereafter referred to as multi-ancestry GWAS) (19). For the downstream analyses, we utilized full summary statistics without applying LD-based clumping. Because these methods incorporate their own LD correction strategies, preclumping was not necessary, enabling us to retain all informative SNPs.

### Post-GWAS Analysis

#### Functional Annotation

We conducted annotation, functional mapping, gene-based analysis, and gene-set analysis of the multi-ancestry GWAS using the SNP2GENE function module of FUMA (20). For gene prioritization, we selected all genes and adopted the default settings otherwise (maximum *p* value of lead SNPs of 5 × 10^−6^; *r*^2^ threshold for independent significant SNPs of 0.6). Subsequently, we used both mapped genes and the significant genes from gene-based analysis to construct a gene expression heatmap via GENE2FUNC function, enabling us to explore the expression of preterm-associated genes across tissues. All analyses were run across ancestries using a multi-ancestry reference panel (1000 Genomes Project phase 3) (21). The GSEA function (FUMA) was used to explore the biological functions of the prioritized genes and compare them with genes in the GWAS Catalog (22) and gene sets in the Molecular Signatures Database (MsigDB) version 7.1 (23). Overall, there were 20,260 background genes applied to GSEA. Gene sets were reported if they met 2 criteria: 1) at least 2 prioritized genes in the gene set, and 2) the adjusted *p* value of the gene set was <.05. Adjusted *p* values for gene set enrichment were computed using the Benjamini-Hochberg false discovery rate (FDR) method, as implemented in FUMA, across all gene sets tested from MsigDB version 7.1 and the GWAS Catalog.

#### Heritability and Genetic Correlations of Preterm Birth and Other Conditions and Traits

To estimate the SNP-based heritability of preterm birth in ASD cases and calculate genetic correlations between preterm birth in our ASD sample and other traits, we used LDSC for analyses involving only EUR ancestry data (EUR-EUR correlations) (24) and Popcorn for analyses involving AMR and AFR ancestry data in comparison to EUR ancestry (AMR/AFR-EUR correlations) (25). While LDSC enabled us to compute genetic correlations across ancestry-matched EUR ancestry individuals in our and external datasets, Popcorn is tailored for cross-population genetic correlation analysis, implementing weighted likelihood function that accounts for the inflation of *z* scores due to LD to provide robust estimates even when LD patterns differ between populations. Although LDSC and Popcorn estimate distinct metrics—genetic correlation and genetic effect correlation, respectively—we interpreted both as indicators of shared genetic architecture for the purpose of comparison across traits and ancestry groups. The traits included in the analyses were epilepsy (26), ADHD (27), anorexia nervosa (28), gastrointestinal disease (29), bipolar disorder (30), schizophrenia (SCZ) (31), musculoskeletal system disease (29), immune system disease (29), and panic disorder (32). All summary statistics were sourced from online databases and subsequently converted to the hg38 genome assembly.

As input for the LDSC analysis, we used summary statistics from a European-ancestry GWAS of preterm birth in the SPARK sample. Prior to the analyses, we filtered SNPs to those found in individuals from the 1000 Genomes Project and converted the files to LDSC format (implemented in munge_sumstats.py). We focused our analysis on well-imputed SNPs by filtering to those found in the HapMap3 reference panel (33). The precomputed LD scores (34), with the major histocompatibility complex (MHC) region excluded, were used for the analyses for individuals of EUR ancestry.

Genotype data for the EUR, AMR, and AFR populations in Popcorn analyses were sourced from the 1000 Genomes Project phase 3 (21). Prior to analysis, standard QC procedures were applied using PLINK, with SNPs with MAF > 0.01, nonautosomal chromosomes, and the MHC region removed. Analyses were performed for both AMR and AFR ancestry individuals, as well as the full meta-analyzed sample. To address the issue of extreme results, the top 5% of betas, which represented significant outliers in the GWAS results, were identified and removed. The distributions of beta coefficients before and after this filtering process are provided in the Supplement (Figure S1), and the changes and the rationale for the exclusion of these outliers are highlighted.

#### Polygenic Risk Score Analyses

To test the genetic overlap between preterm birth and other traits in our sample, we 1) tested the association between a polygenic risk score (PRS) for preterm birth derived from a GWAS conducted in the general population and other phenotypes available for individuals in the SPARK cohort, and 2) calculated PRSs for additional phenotypes (both psychiatric and nonpsychiatric) in the SPARK sample and tested their association with the preterm birth status reported for the SPARK participants.

To compute the PRS across the traits and ancestries, we used BridgePRS (35), an advanced Bayesian PRS method tailored to enhance the transferability of PRS across diverse ancestral backgrounds. Each ancestry was analyzed separately to account for ancestry-specific genetic architectures. Clumping was performed with parameters set to 1000 kb and an LD *r*^2^ threshold of 0.01 to ensure the independence of SNPs. The LD reference panel used was based on 1000 Genomes variants with an MAF >1% from the corresponding population. Additionally, the analysis included the first 15 PCs as covariates.

#### PRS Analyses: Associations Between Polygenic Liability to Preterm Birth and Other Phenotypes

We analyzed the association between polygenic liability (PRS) to preterm birth and an array of medical conditions captured through the medical screening module in SPARK. A preterm birth PRS was estimated for all autistic individuals in our analyses, irrespective of preterm status, using external preterm base data (EUR) (*N* = 84,689) (15) to avoid inflating the associations between PRS and other phenotypes. Medical history module data in SPARK is based on self- or parental report of clinical diagnosis and is divided into several domains. A positive reply for each domain is followed by more specific and detailed questions regarding the conditions within that domain. We tested the association between the preterm PRS and 68 medical conditions in the domains of 1) birth or pregnancy complications, 2) attention or behavioral disorders, 3) developmental disabilities, 4) growth conditions, 5) neurological conditions, 6) vision or hearing conditions, 7) psychiatric disorders, and 8) sleep, feeding, eating, or toileting problems. Each of the medical conditions within these domains served as an outcome in a separate model. The PRS score was standardized prior to analyses. Additional model covariates included the individual’s sex and year of birth. Given the preimputation QC, the individuals were unlikely to be genetically related; however, to address the potential nongenetic correlation between individuals from the same family, the standard errors were estimated using a clustered sandwich estimator, implemented in the clusterSEs package (version 2.6.2). To correct for multiple testing, FDR correction was applied to all *p* values.

#### PRS Analyses: Association Between Preterm Birth Status and Polygenic Liability to Other Traits

PRSs for additional traits were calculated in BridgePRS using relevant summary statistics from EUR, AFR, and AMR ancestries, as available. Details on the datasets used for each trait and ancestry are provided in Table S1.

## Results

Genotype data for 647,280 variants from 69,758 individuals with genotype data (56.8% male) were available from the SPARK project (36). We excluded 32,884 individuals without a confirmed ASD diagnosis. After the genotyping QC, we retained 400,266 variants in 31,947 individuals, including 3308 autistic individuals born prematurely and 28,639 born at term. After imputation, there were a total of 8,855,081 variants with MAF > 0.01 and imputation quality score (INFO) > 0.5 used in the analyses.

### Genetic Differences Between ASD Cases Born Term and Preterm

Classification into 3 ancestry groups provided the best fit for the data, and individuals who were highly admixed were excluded from the analyses, leaving 2888 preterm cases in the analysis. We conducted 3 ancestry-specific GWASs (AFR: *n*_preterm_ = 196, *n*_term_ = 1722; AMR: *n*_preterm_ = 396, *n*_term_ = 3955; EUR: *n*_preterm_ = 2296, *n*_term_ = 19,173) adjusting for the 15 PCs.

In ancestry-specific GWASs, only the AFR GWAS had SNPs reaching the genome-wide level of significance (*p* < 5.0 × 10^−8^; rs117965482 [chr 12], *p* = 3.04 × 10^−8^ and rs78395263 [chr 12], *p* = 3.89 × 10^−8^) (Figure S2). The top associated variant from the AMR GWAS was rs4713424 (chr 6, *p* = 1.30 × 10^−7^ (Figure S3), and in the EUR GWAS, it was rs114444 (chr 16, *p* = 1.45 × 10^−7^) (Figure S4). The direction of the effects of those loci on preterm birth differed across ancestries (e.g.*,* rs78395263 [effect allele T] had an allele frequency of 3.6% in EUR and 1.6% in AFR, with EUR-odds ratio [OR] = 0.998, *p* = .978 and AFR-OR = 5.40, *p* = 3.89×10^−8^; rs117965482 [effect allele C] had an allele frequency of 0.4% in EUR and 0.5% in AFR, with EUR-OR = 0.992, *p* = .920 and AFR-OR = 5.50, *p* = 3.04 × 10^−8^) (Figure S5). The quantile-quantile plots for each GWAS can be found in Figure S6.

**Figure 1:**
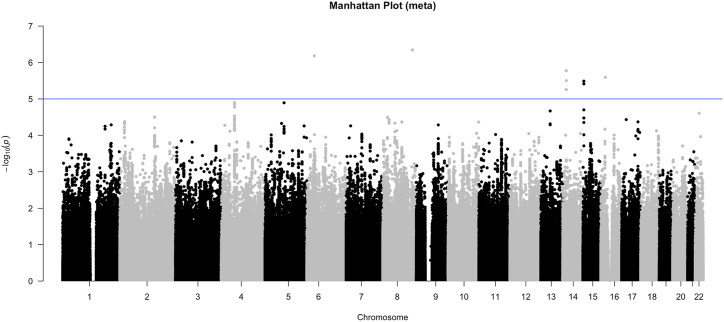
Manhattan plot of the results from the logistic regression of the meta-analysis of preterm (*n* = 3308) vs. nonpreterm (*n* = 28,639) autism spectrum disorder cases, after adjustment for the first 15 principal components. Genomic regions contain single nucleotide polymorphisms that exceed 5 × 10^−05^ above the blue line. No locus in the meta-analyzed genome-wide association study was genome-wide significant.

The differential direction of the effect sizes rendered the variants identified in the ancestry-specific GWAS nonsignificant in the meta-analysis. The top associated variant in the meta-analysis was rs2019941, effect allele T (OR = 0.71 [0.62–0.81], *p* = 4.52 × 10^−07^) on chromosome 8 (Figure 1).

#### Functional Annotation of the Preterm-Associated Loci in ASD

In FUMA analyses of the multi-ancestry meta, we identified 4 genomic risk loci associated with preterm birth in ASD cases at *p* < 5.0 × 10^−6^, located on chromosomes 8, 14, 15, and 16 (Figure S7). These loci include the SNPs rs2019941 (chr8:129126122, *p* = 4.52 × 10^−7^), rs118182680 (chr14:35462315, *p* = 1.67 × 10^−6^), rs58406701 (chr15:28114815, *p* = 3.24 × 10^−6^), and rs114444 (chr16:19609590, *p* = 2.54 × 10^−6^). After LD pruning, 4 independently significant SNPs were retained. Additionally, 59 candidate SNPs were identified via positional mapping, 12 of which were correlated with previously reported loci through LD, suggesting a shared genetic architecture with related traits. However, no direct trait annotations for these loci were available in the GWAS Catalog, suggesting that their functional significance in preterm birth and ASD has not been established. Most candidate SNPs were intronic, supporting a potential role for noncoding regulatory mechanisms in preterm birth susceptibility (Figure S8). Functional annotation based on positional mapping identified 3 genes related to preterm birth in ASD (*SRP54*, *C16orf62*, and *OCA2*).

Tissue enrichment analysis of these 3 genes using MAGMA revealed nominally significant enrichment in nerve tibial (*p* = .043) and artery aorta (*p* = .047) tissues among 53 specific tissue types (Figure S9A). When grouped into 30 general tissue types, the results revealed significant enrichment in the nerve tissue (*p* = .035), as well as reproductive tissues such as the uterus (*p* = .038) and ovaries (*p* = .045) (Figure S9B).

To further investigate tissue-specific gene expression, we analyzed differential gene expression patterns using GTEx version 8 across 30 general and 54 specific tissue types. The analysis did not reveal any tissues exhibiting statistically significant differential expression after multiple testing correction. The highest level of gene expression enrichment was in the testis. A heatmap of average expression levels for *SRP54*, *C16orf62*, and *OCA2* across various tissues highlighted distinct tissue-specific expression patterns. SRP54 exhibited high levels of tissue-specific expression, whereas *OCA2*, on the other hand, showed relatively uniform expression levels across tissues. *C16orf62* showed a range of expression levels across tissue types (Figure S10).

### Genetic Overlap Between Preterm Birth and Other Phenotypes

The SNP-based heritability of preterm birth in the full, multi-ancestry sample calculated with LDSC was significant and higher than reported in the general population (14.24%, SE = 0.023, mean χ^2^ = 1.04), compared with 2.5% reported in the general population (15) (see Figure S11 for heritability estimates across ancestries). The SNP-based heritability of preterm birth in the EUR ancestry sample calculated with LDSC was statistically significant (35.4%, SE = 0.027, mean χ^2^ = 1.03).

The genetic correlation between preterm birth in our ASD sample and an external sample drawn from the general population was nonsignificant, both in the full, multi-ancestry sample (*r*_g_ = −0.099, *p* = .720) and the EUR ancestry (*r*_g_ = 0.105, *p* = .548) subset of the SPARK individuals.

### Genetic Correlations

**Figure 2:**
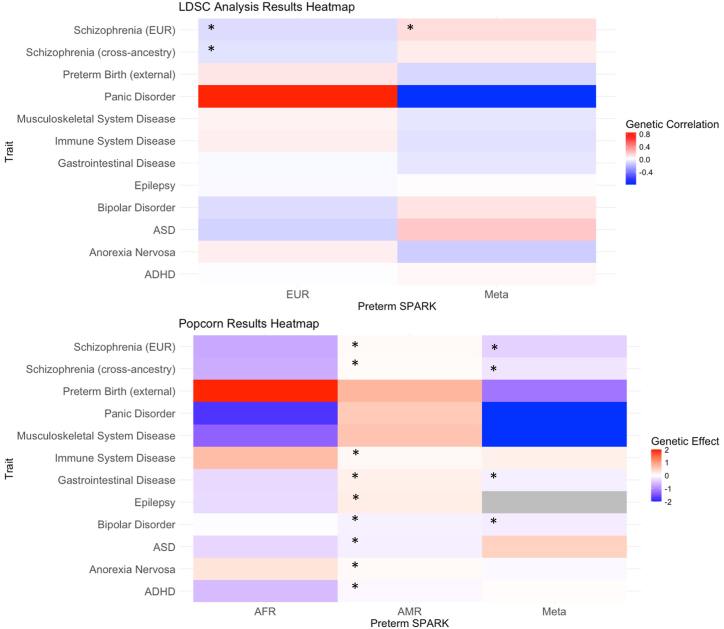
Heatmap illustrating the genetic correlations and effects between traits as estimated using LDSC and Popcorn analyses. Each cell represents the correlation and effect between 2 traits, with the color gradient ranging from blue (low correlation) to red (high correlation). Significant genetic correlations and effects are indicated by an asterisk. To improve the visual clarity of the heatmap, the color scale was adjusted by clipping genetic effects outside the range of −2 to 2. ADHD, attention-deficit/hyperactivity disorder; AFR, African/African American; AMR, admixed American; ASD, autism spectrum disorder; EUR, non-Finnish European; SPARK, Simons Foundation Powering Autism Research for Knowledge.

Genetic correlations between preterm birth in our ASD sample and other traits estimated for the meta-analyzed and EUR ancestry individuals in the LDSC analysis were all nonsignificant except for a negative correlation with SCZ (EUR) (Table S2 and Figure 2). However, in the meta-analyzed sample analyzed with Popcorn, 4 of 12 genetic correlations between preterm birth in ASD cases and other traits were significant, all of them negative. The strongest correlations included those between preterm birth and SCZ (cross-ancestry) (*p* = .001), SCZ (EUR) (*p* = .002), bipolar disorder (*p* = .007), and gastrointestinal disease (*p* = .025) (Figure 2 and Table S5). Ancestry-specific genetic correlations are presented in Tables S3 and S4.

### PRS Analyses

#### Polygenic Liability to Preterm Birth and Reported Medical Conditions

Polygenic liability to preterm birth (PRS) computed using BridgePRS was nominally significantly associated with 4 medical conditions reported in the SPARK medical screening, including birth defects and mood or psychiatric conditions (Figure S11 and Table S6). After adjusting for multiple testing, 3 conditions, including missing kidney (OR = 2.36; 95% CI, 2.24–2.48), esophageal atresia (OR = 0.69; 95% CI, 0.67–0.70), and missing uterus (OR = 0.41; 95% CI, 0.39–0.42), remained statistically significant, although the direction of the effects differed across these associations.

#### Preterm Birth and Genetic Liability to Medical Conditions

Reported preterm birth status was not significantly associated with genetic liability to any of the additional traits that we analyzed in any of the ancestry-specific analyses (Tables S7 and S8 and Figure S13).

## Discussion

We examined the genetic underpinnings of preterm birth in autistic individuals, building on our previous work demonstrating clinical heterogeneity among them in association with different early-life exposures (4). Our results demonstrate that the differences in comorbid conditions in individuals diagnosed with ASD born preterm versus term co-occur with genetic differences between those groups, highlighting the potential of integrating genetic and early-life information to understand autism heterogeneity.

Our findings revealed ancestry-specific SNPs significantly associated with preterm birth (rs78395263 and rs117965482 for AFR); however, none of these associations remained significant in the trans-ancestry meta-analysis. This suggests that genetic effects on preterm birth may vary between populations, as suggested previously (37,38), and that combining data from diverse ancestries could weaken these effects (39,40). It is possible that these genetic factors operate through environmental influences in an ancestry-specific manner, as reported previously for other traits (41). While such environment-mediated effects could be largely maternal in origin, we were not able to separate distinct maternal and fetal effects on the risk of preterm birth (15,42). However, given the small sample size of the AFR-specific GWAS, these findings should be interpreted with caution because they may reflect statistical artifacts such as the winner’s curse rather than true ancestry-specific effects.

The gene regions mapped to significant SNPs through positional mapping—*SRP54*, *OCA2*, and *C16orf62* (also called VPS35L)—did not reach genome-wide significance in gene-based tests. These genes have previously been linked to congenital neutropenia (43) (*SPR54*), pigmentation and vision (44) (*OCA2*), and Ritscher-Schinzel syndrome (45,46), a neurodevelopmental condition associated with a host of comorbid conditions, including alterations in the skeletal, gastrointestinal, immune, and genitourinary systems (47).

Leveraging the genome-wide signals in the SPARK and general population samples, we observed that the genetic correlation of preterm birth in individuals with ASD and the general population (15) was not significant, indicating that the genetic factors contributing to preterm birth may differ in individuals with autism. While the GWAS signal in autistic individuals may be enriched for pleiotropic loci—affecting both autism and preterm birth risk—compared with the signal in the general population, the power to uncover a genetic correlation between those two was limited by the low SNP heritability, warranting further replication. Alternatively, our preterm in ASD GWAS could be picking up general differences in the genetic architecture between autistic individuals born preterm and those born at term, even if those differences are not directly related to preterm birth. For example, given independent, additive etiological effects of genetic variation and preterm birth on autism (48), autistic individuals born preterm may carry fewer common autism-associated variants than those born at term, creating an apparent negative association in the GWAS that reflects group differences rather than true pleiotropic effects.

The low SNP heritability estimates of preterm birth reported in our and previous studies stand in contrast to twin- and family-based estimates ranging from 30% to 40% (49), likely indicating the effects of rare variants not captured by common SNPs. This discrepancy likely reflects the contribution of rare variants not captured by standard SNP arrays, which can result in underestimation of heritability when using GWAS data (41,50,51). Alternatively, heritability may be inflated in family-based studies due to shared environmental influences or nonadditive genetic effects (50).

Further analyses revealed genetic overlap between preterm birth and other traits and conditions in autistic individuals, with differences across ancestry groups. This may reflect ancestry-specific variants (37,38), distinct LD patterns in AMR and AFR populations (52), or environmental and population-specific interactions. In the meta-analyzed sample, we found significant genetic correlations with SCZ (EUR) using LDSC, as well as SCZ (both EUR and cross-ancestry), gastrointestinal disease, and bipolar disorder using Popcorn. The negative genetic correlation with SCZ is notable because SCZ risk variants are typically overtransmitted in autism (53), and an observational association exists between SCZ and preterm birth (54). This pattern may reflect variants with opposite effects on SCZ and preterm birth, lower genetic liability to autism in preterm individuals (48), and/or the indirect (maternal) genetic effects acting through prenatal environment. The differences between LDSC and Popcorn results could be attributed to how each method estimates genetic association; LDSC estimates genetic correlation based on within-population LD structure and sample size, while Popcorn accounts for ancestry-specific LD and genetic architecture across populations. Lower heritability in some subgroups may also reduce LDSC’s power to detect correlations.

Finally, PRS analyses offered us an independent opportunity to evaluate the genetic overlap between preterm birth in ASD and other phenotypes. Using BridgePRS (35), we evaluated polygenic risk across traits in all ancestry groups. Our results highlight that polygenic liability to preterm birth is associated with co-occurring conditions, especially birth defects. However, some of these associations were in the opposite direction than expected—we observed lower risk of some birth defects in individuals with higher preterm PRSs despite the higher frequency of congenital problems in preterm individuals in the general population (55), indicating that caution is warranted when interpreting these associations. Additionally, the lack of association between the preterm birth PRS and preterm birth status in the SPARK sample suggests that the genetic factors contributing to preterm birth may differ in individuals with ASD compared with the general population. This is consistent with our finding that the genetic correlation between preterm birth in ASD and in the general population was not significant, further supporting the idea that distinct genetic mechanisms may underlie preterm birth in individuals with autism.

### Conclusions

We utilized a large and diverse cohort of autistic individuals with comprehensive medical and genotype data. By applying ancestry-specific methods, we maximized sample inclusion and uncovered both phenotypic and genetic associations that suggest that early-life exposures may contribute to clinical heterogeneity in autism. Our results underline the importance of considering ancestry-specific genetic factors when studying complex traits such as preterm birth in ASD. However, while our study supports a proof-of-principle concept—that early-life exposure data can be used to probe genetic differences between autistic individuals and map those differences onto phenotypic heterogeneity—the power in our study was limited, likely due to the complex, rare variant-driven architecture of preterm birth (56, 57, 58). Additionally, we were not able to disentangle direct and indirect (maternal) genetic effects in our GWAS. Future studies should aim to replicate these findings in larger cohorts, accounting for both common and rare variation, including structural variants (58).

## Supplementary Material

Figures S1–S13 and Tables S1–S8

Key Resources Table
