# Supplementary material for: Autism Heterogeneity Related to Preterm Birth: Multi-Ancestry Results From the Simons Foundation Powering Autism Research for Knowledge Sample
Source: Biol Psychiatry Glob Open Sci. 2025 Sep 17;6(1):100614. doi: 10.1016/j.bpsgos.2025.100614 (PMC12662992; doi:10.1016/j.bpsgos.2025.100614)
Supplement: Figures S1–S13 and Tables S1–S8 [file mmc1.pdf]

SUPPLEMENTARY INFORMATION

Autism Heterogeneity Related to Preterm Birth: Multi-Ancestry Results From the SPARK sample

Chatzigeorgiou *et al.*

Supplemental Figures

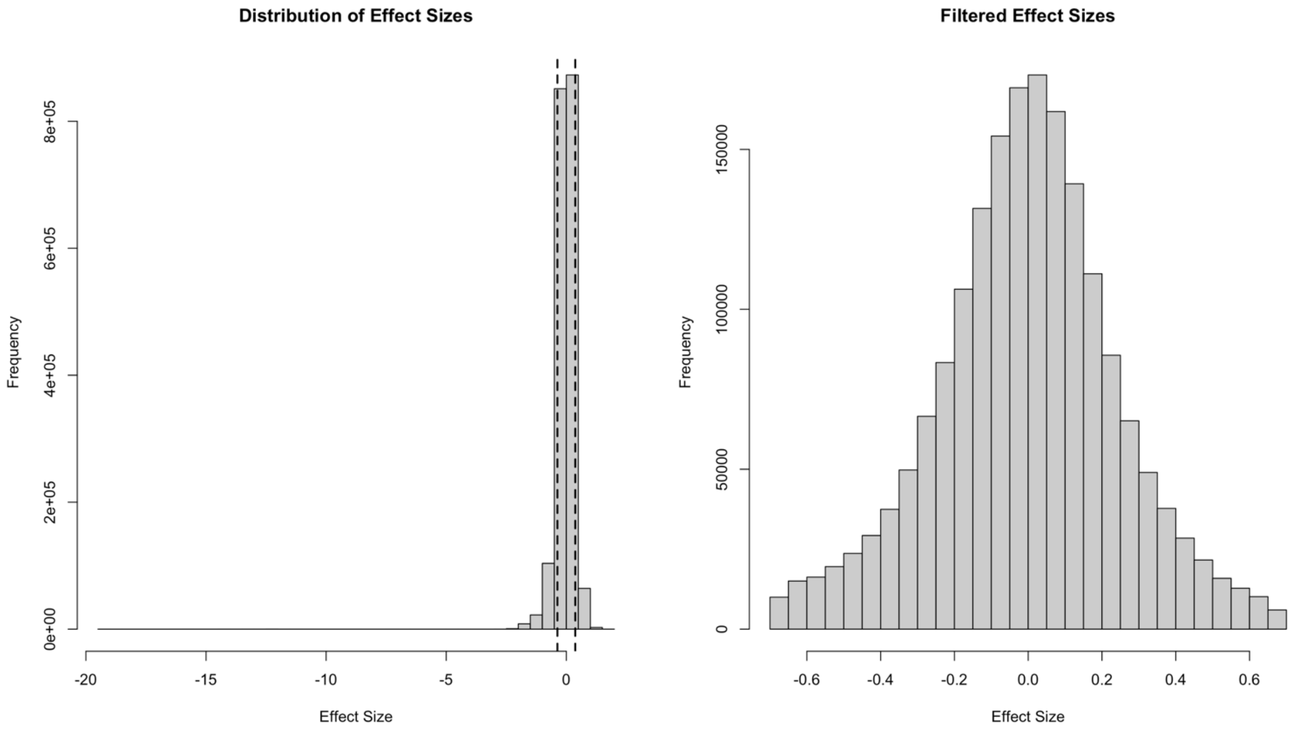

Fig. S1. The distribution of effect sizes is shown before filtering (left panel) and after filtering (right panel). In the left panel, the wide range of effect sizes includes extreme outliers. A dashed line indicates the cut-off value of 0.69, corresponding to the top 5% of effect sizes, which were removed during filtering. After filtering (right panel), the distribution exhibits a more symmetrical and centered pattern, highlighting the removal of outliers and the refined focus on relevant effect sizes for downstream analyses.

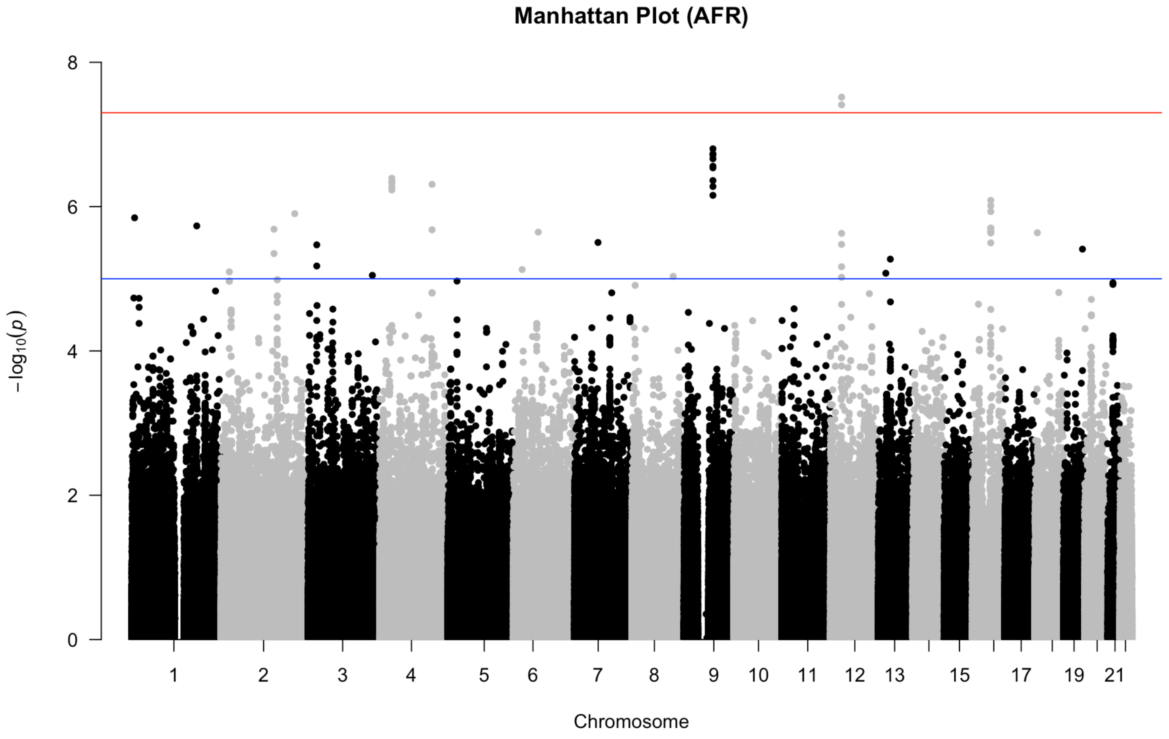

Figure S2. Manhattan plot of the results from the logistic regression of preterm (n=196) vs. non-preterm (n=1,722) ASD cases of African/African-American ancestry, after adjustment for the first 15 ancestry-specific PCs. Genomic regions contain SNPs that exceed i) the genome-wide significance threshold of 5×10^{-08} are shown above the red line and ii) 5×10^{-05} above the blue line.

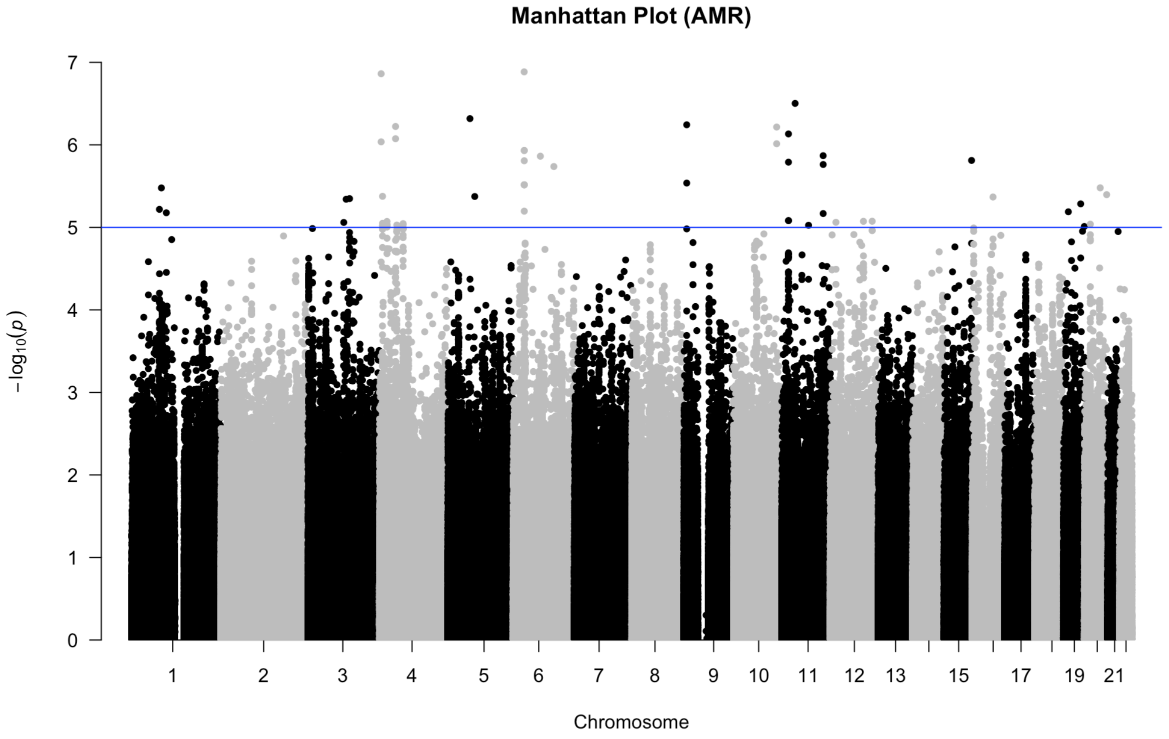

Figure S3. Manhattan plot of the results from the logistic regression of preterm (n=396) vs. non-preterm (n=3,955) ASD cases of Admixed American ancestry, after adjustment for the first 15 ancestry-specific PCs. Genomic regions contain SNPs that exceed 5×10^{-05} above the blue line. No locus in AMR GWAS was genome-wide significant.

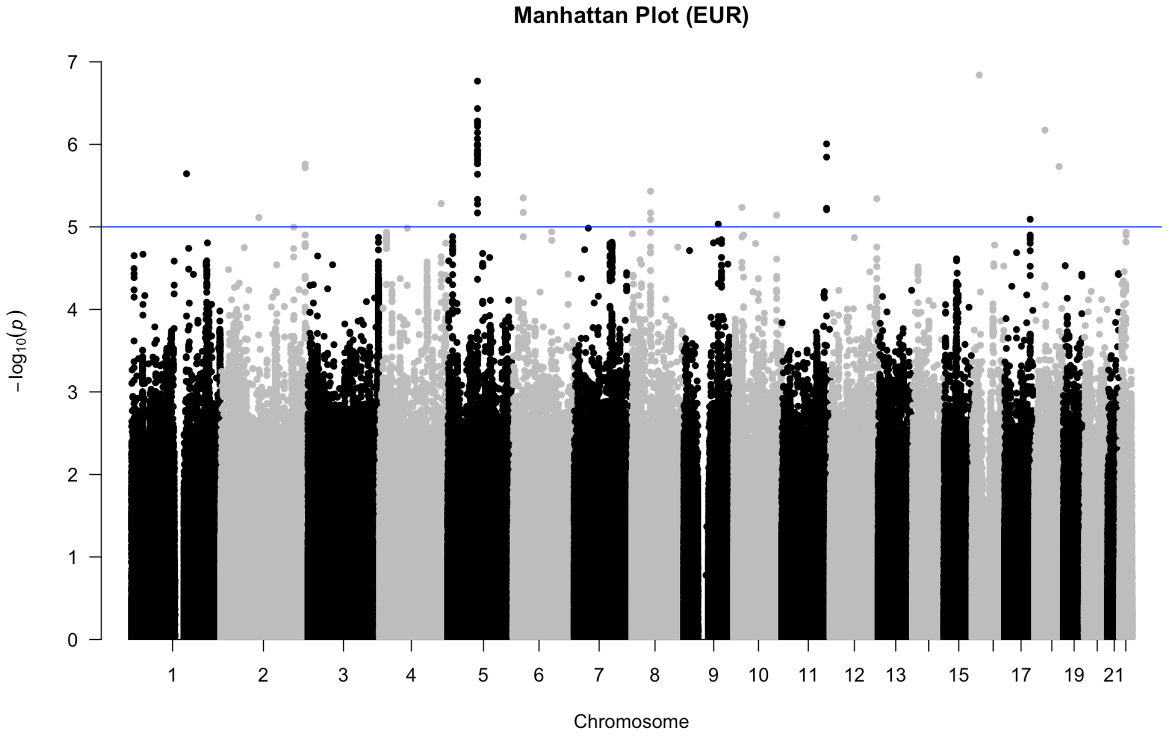

Figure S4. Manhattan plot of the results from the logistic regression of preterm (n=2,296) vs. non-preterm (n=19,173) ASD cases of Non-Finish European ancestry after adjustment for the first 15 ancestry-specific PCs. Genomic regions contain SNPs that exceed 5 x 10^{-05} above the blue line. No locus in EUR GWAS was genome-wide significant.

A)

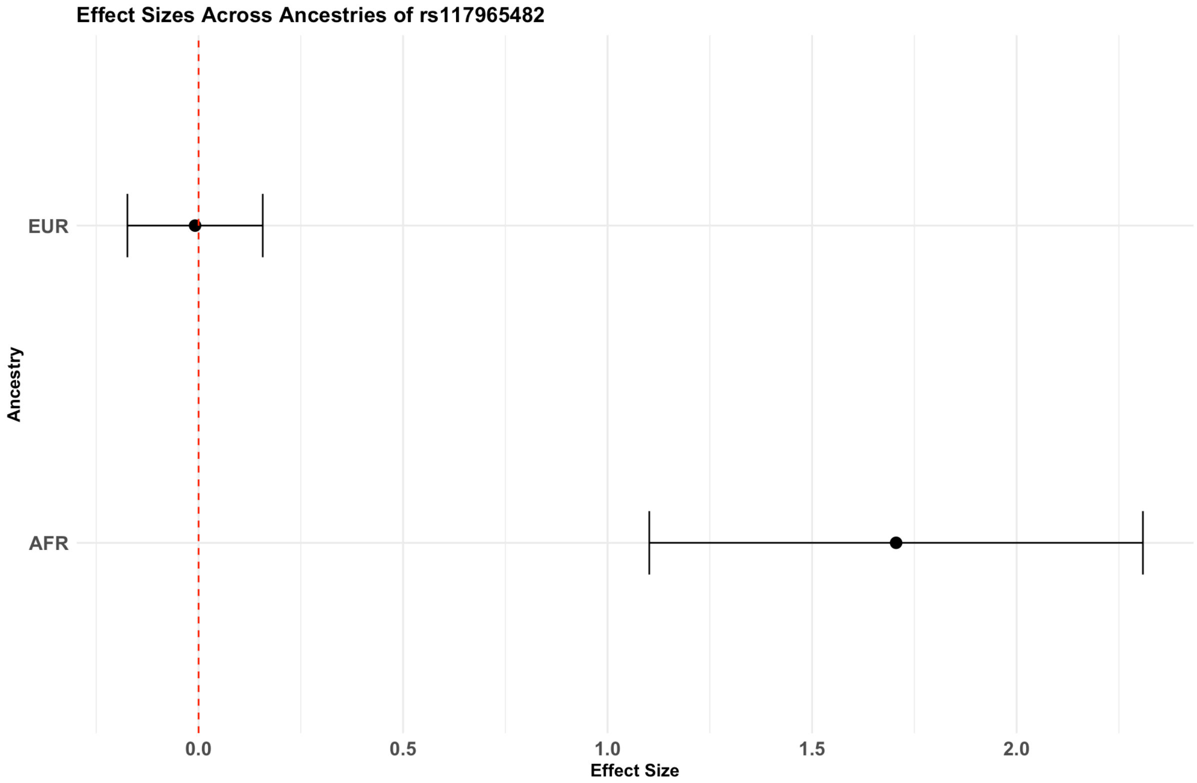

B)

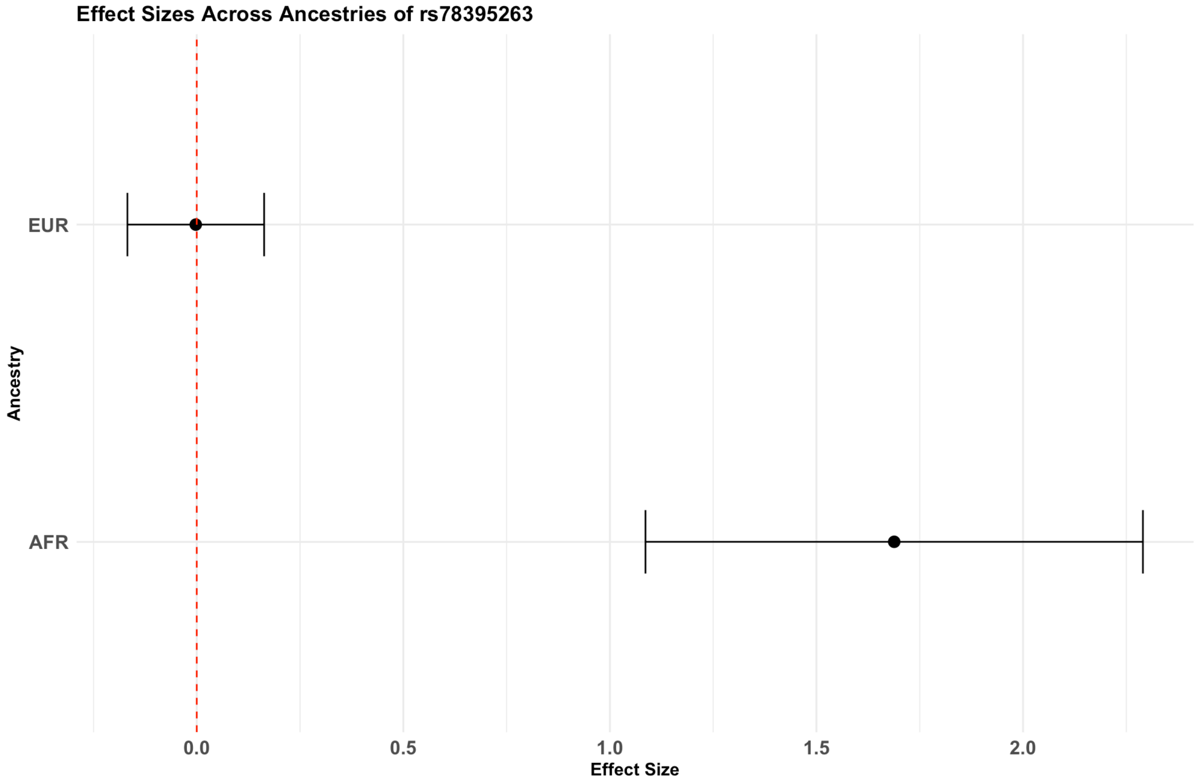

Fig. S5. Comparison of Effect Sizes Across EUR and AFR Populations for genome-wide significant SNPs. This figure presents the effect sizes of two genome-wide significant SNPs across European (EUR) and African (AFR) ancestry groups. Panel A shows the effect size distribution for SNP rs117956482 (effect allele C), while Panel B illustrates the effect size for SNP rs78395263 (effect allele T). The x-axis represents the effect size (beta), with error bars indicating confidence intervals. The red dashed line marks the null effect for comparison. The AMR ancestry and meta-analysis did not contain these SNPS.

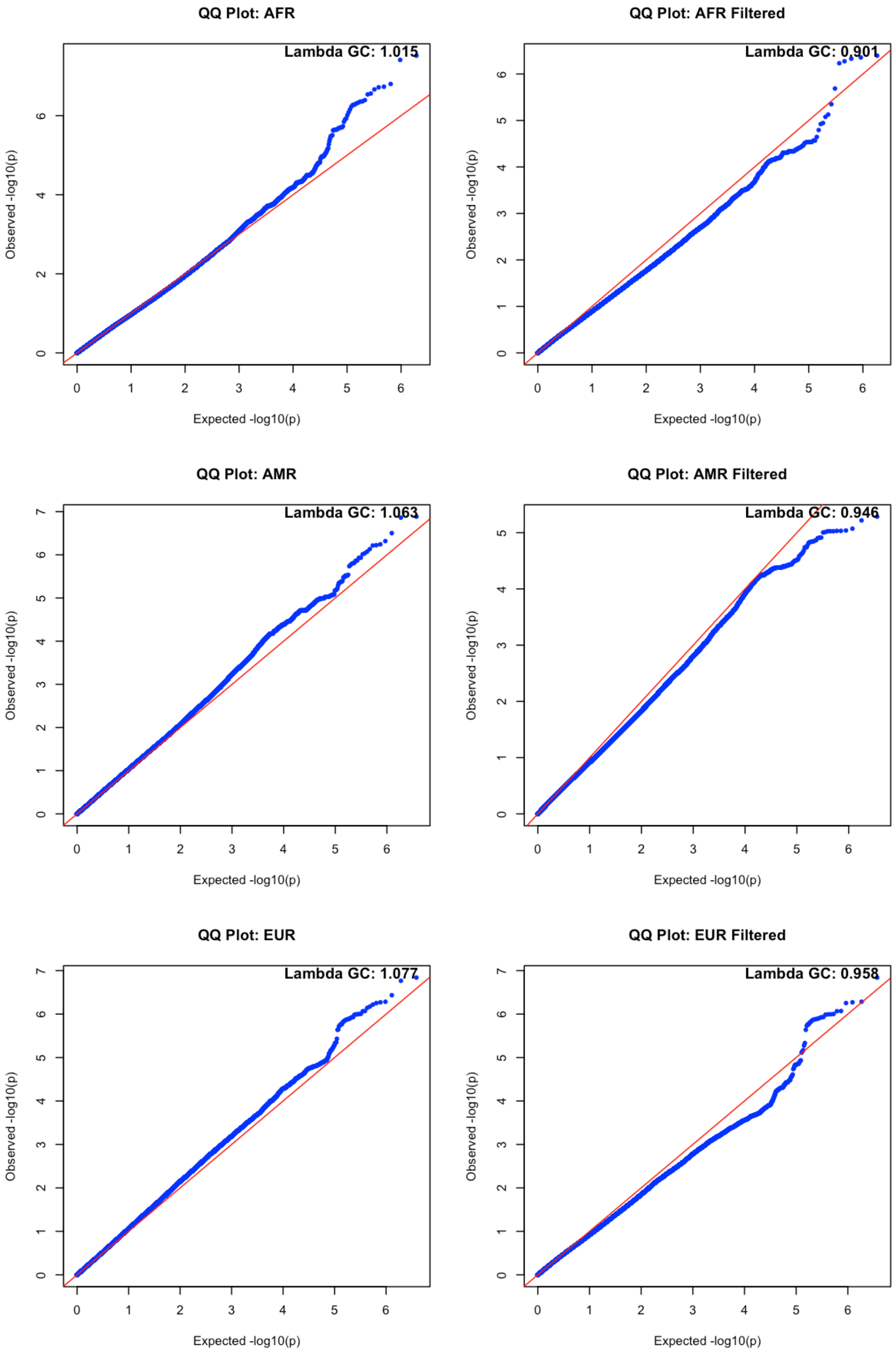

Fig S6. QQ plots of ancestry specific GWAS both before and after filtering. The red diagonal line represents the expected distribution, while deviations indicate potential inflation or deflation of test statistics. The genomic control factor (Lambda GC) is reported for each dataset to assess overall inflation.

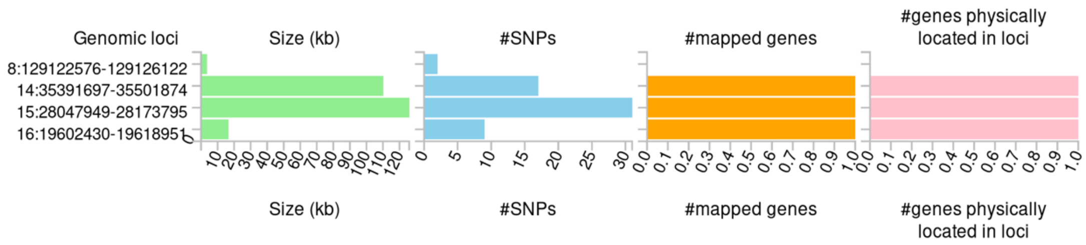

Fig S7. Summary of genomic risk loci analysis based on GWAS of ASD preterm individuals. Genomic risk loci are displayed by the 'chromosome:start position-end position' on the Y axis. Histograms from left to right depict the size of the genomic locus, number of candidate SNPs in the genomic locus, number of mapped genes by positional mapping and eQTL mapping in the genomic locus, and the number of genes known to be located within the genomic loci, respectively.

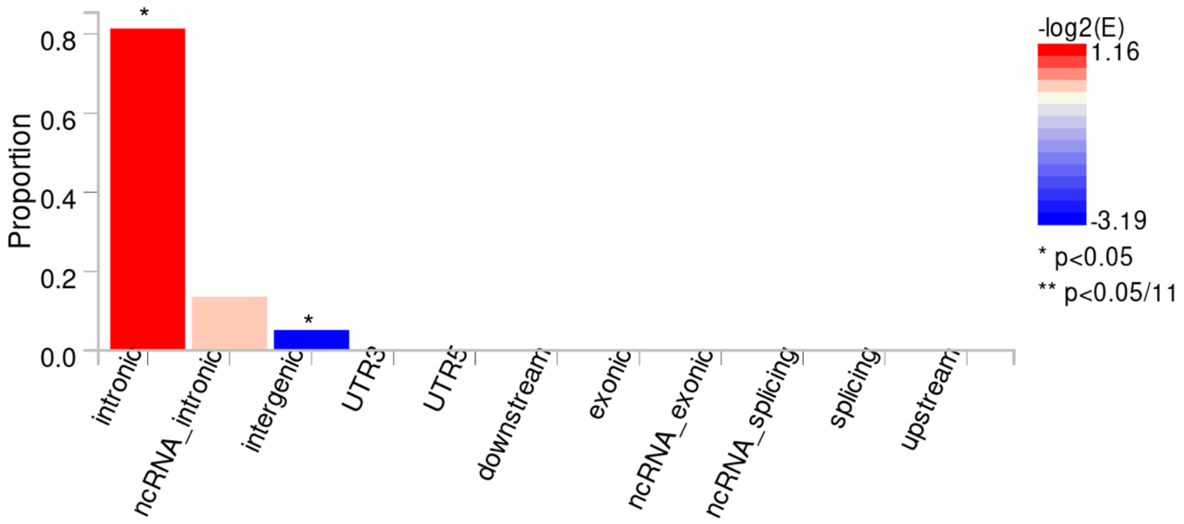

Fig S8. Functional consequences of 59 SNPs on genes. The plot shows the proportion of SNPs located in various genomic regions. The color gradient represents the -log2 enrichment (E) of SNPs in each category, with red indicating enrichment and blue indicating depletion. Asterisks denote statistically significant categories (*p < 0.05, **p < 0.05/11).

A)

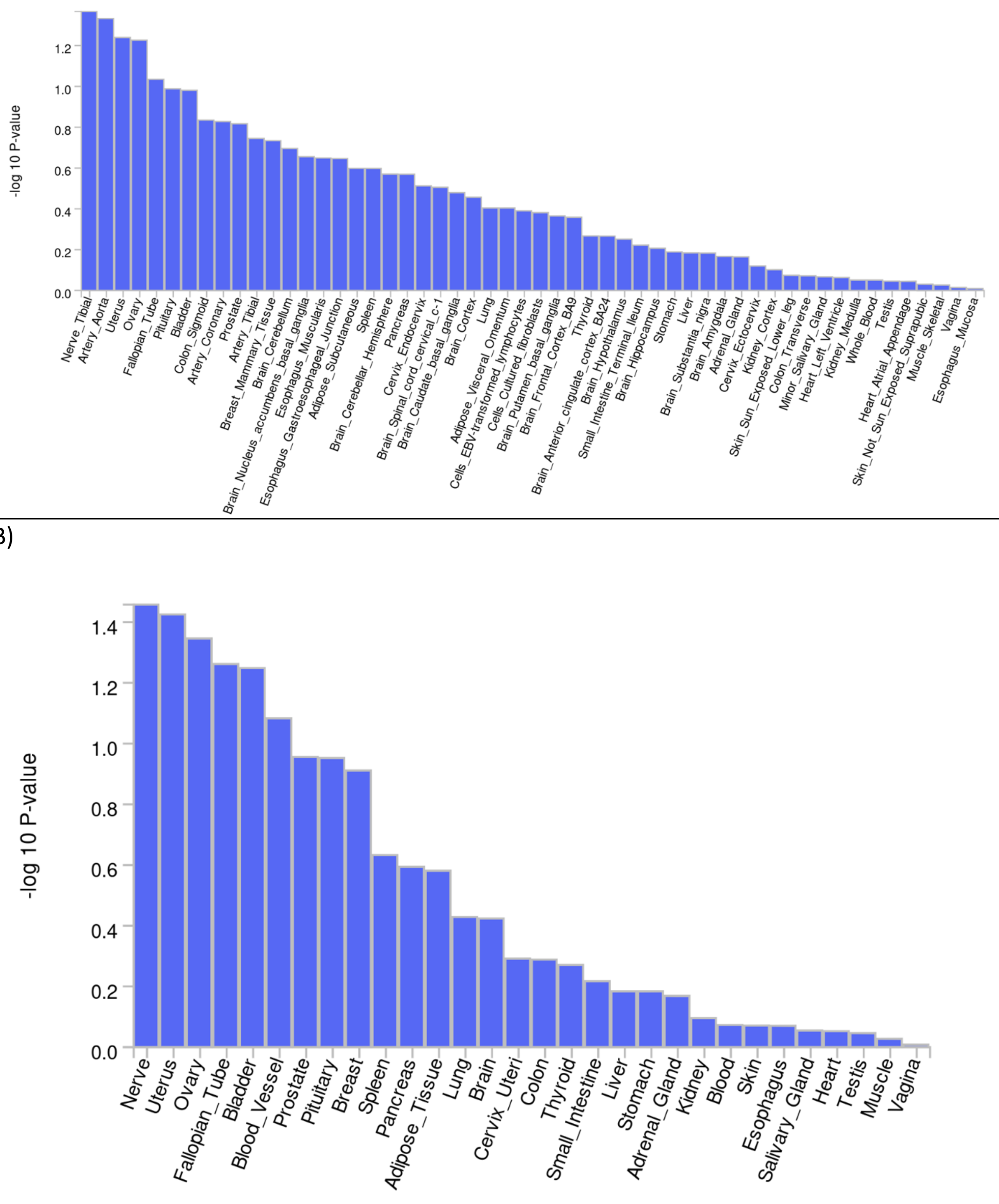

Figure S9. MAGMA Tissue Expression Analysis. Panel A displays the association of 53 specific tissue types with the analyzed trait based on -log10(p-values), indicating their relative enrichment. Panel B summarizes these findings across 30 general tissue types, highlighting broader tissue categories with significant associations. The x-axis represents tissue types, while the y-axis shows statistical significance (-log10 p-value) for their involvement.

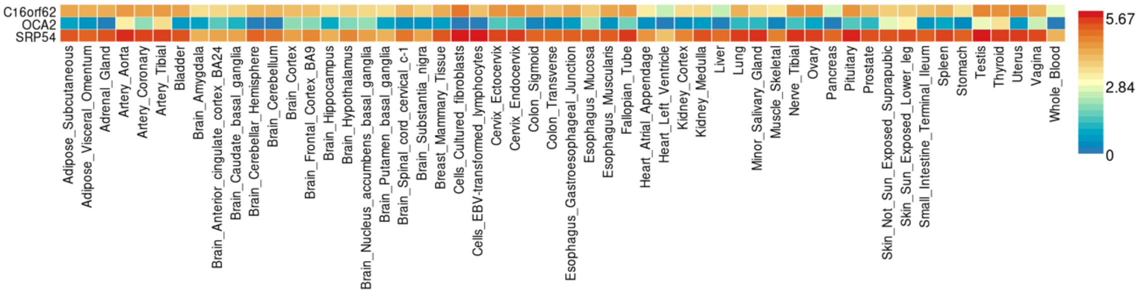

Fig. S10. Heat map of tissue-specific gene expression of genes corresponding to genetic variants from meta-analysis of pre-term and term ASD individuals. The heat map is plotted using FUMA v1.5.0 and gene expression data from GTEx v8 54 tissue types. The heat map was ordered by both gene and tissue clustering. Darker red represent higher expression of that gene compared to darker blue color across genes and tissues.

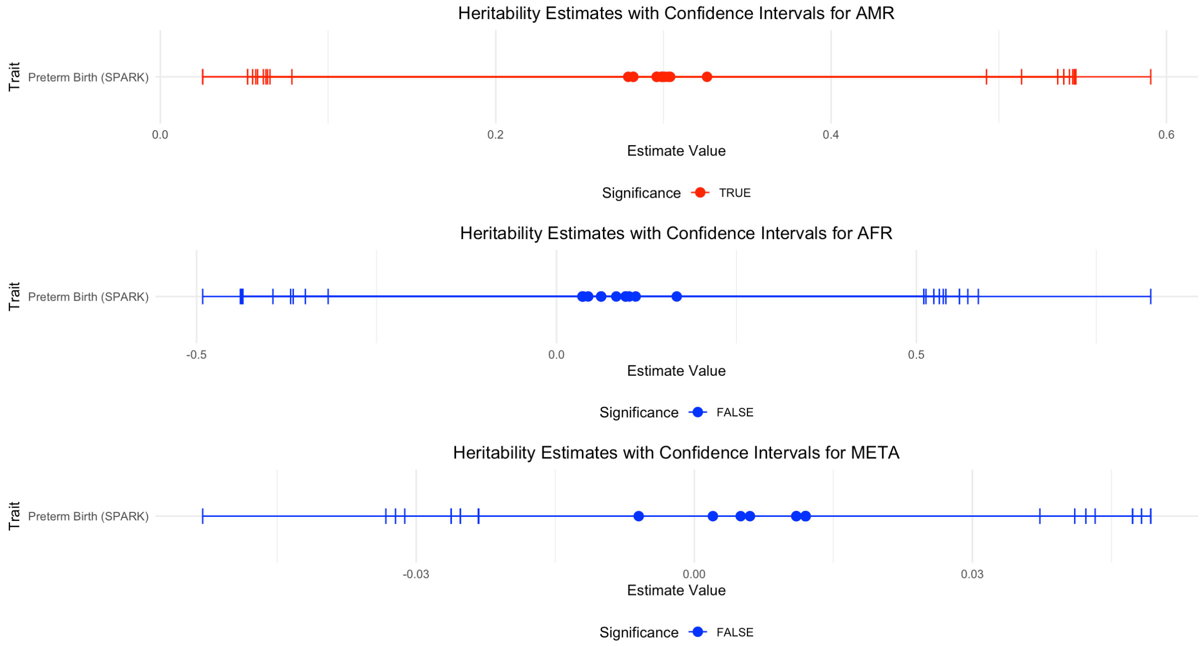

Figure S11. Heritability estimates for Preterm birth in the individuals of AMR (A), AFR (B) ancestry and the meta-analysis (C). Significance is indicated by red dots, while blue dots represent non-significant results with its corresponding confidence intervals. Preterm birth (SPARK) has multiple heritability estimates because its heritability was calculated multiple times using different models, subsets of individuals, or through repeat runs. This variability can occur when analyzing the same data with slightly different assumptions or subsets. However, even though these estimates are different, they are close to each other, indicating consistency in the heritability estimates despite the use of varying methods or data subsets.

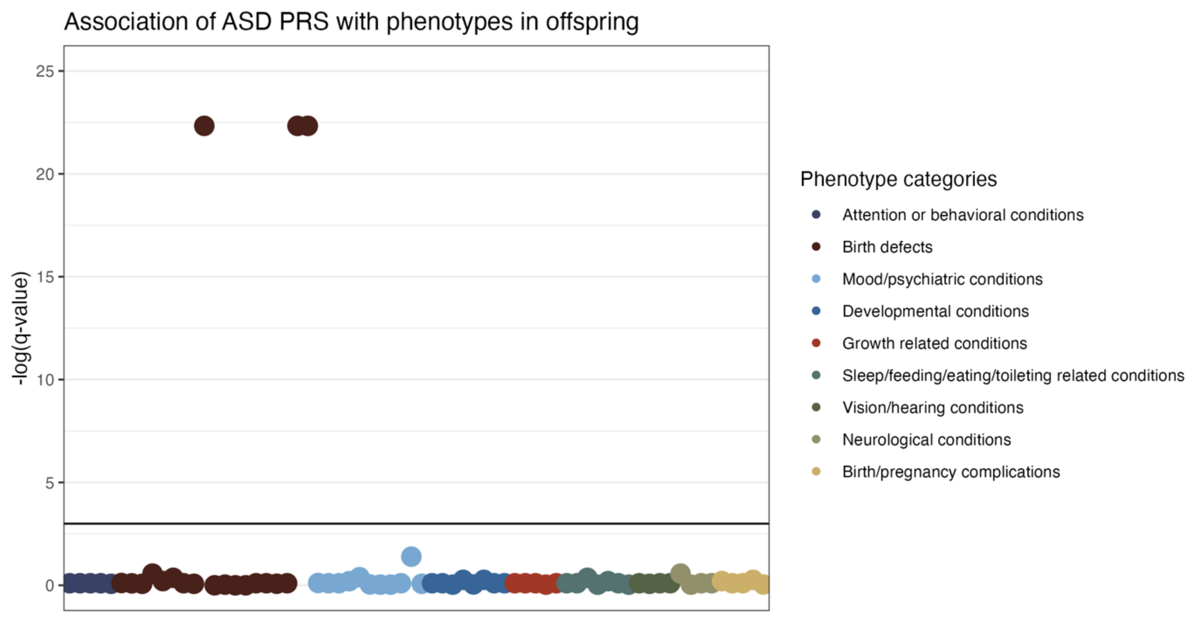

Figure S12. Associations between polygenic liability to preterm birth (PRS) and various medical conditions (see Table S5 for regression parameters).

A)

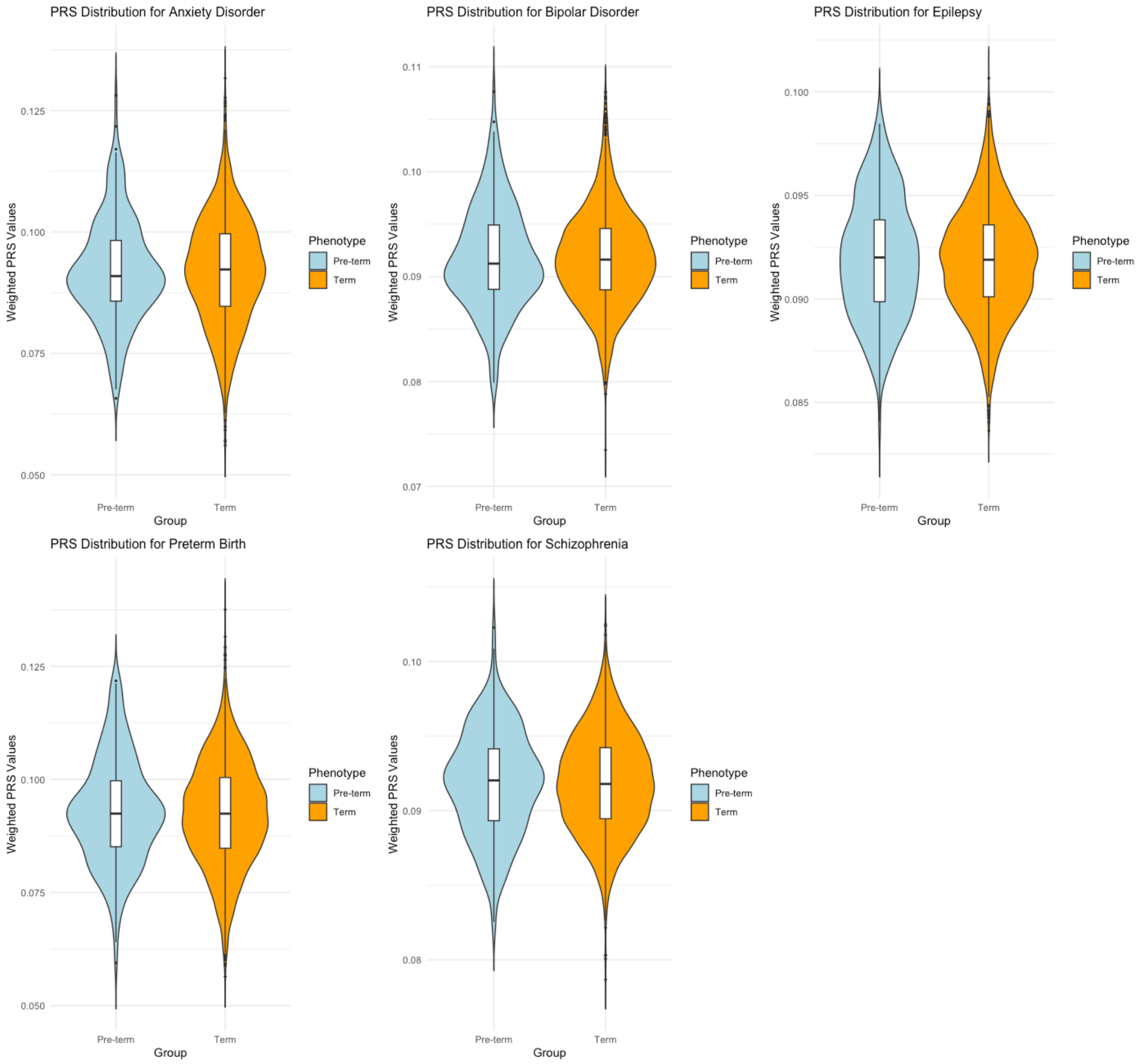
PRS Distribution for Anxiety Disorder
Weighted PRS Values
0.125
0.100
0.075
0.050
Pre-term
Term
Group
Phenotype
Pre-term
Term
PRS Distribution for Bipolar Disorder
Weighted PRS Values
0.11
0.10
0.09
0.08
0.07
Pre-term
Term
Group
Phenotype
Pre-term
Term
PRS Distribution for Epilepsy
Weighted PRS Values
0.100
0.095
0.090
0.085
Pre-term
Term
Group
Phenotype
Pre-term
Term
PRS Distribution for Preterm Birth
Weighted PRS Values
0.125
0.100
0.075
0.050
Pre-term
Term
Group
Phenotype
Pre-term
Term
PRS Distribution for Schizophrenia
Weighted PRS Values
0.10
0.09
0.08
Pre-term
Term
Group
Phenotype
Pre-term
Term

B)

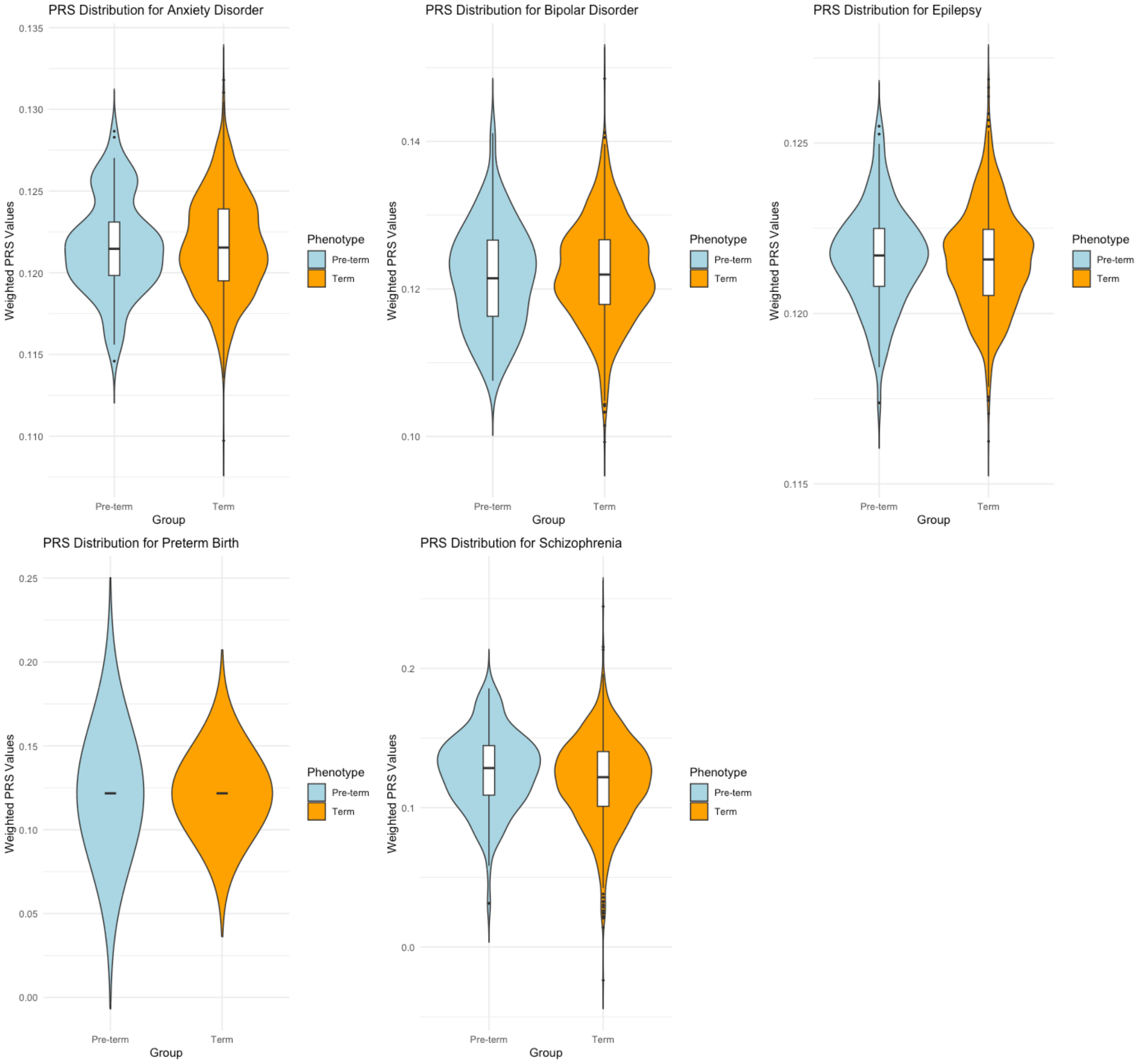
PRS Distribution for Anxiety Disorder
Weighted PRS Values
0.135
0.130
0.125
0.120
0.115
0.110
Pre-term
Term
Group
Phenotype
Pre-term
Term
PRS Distribution for Bipolar Disorder
Weighted PRS Values
0.14
0.12
0.10
Pre-term
Term
Group
Phenotype
Pre-term
Term
PRS Distribution for Epilepsy
Weighted PRS Values
0.125
0.120
0.115
Pre-term
Term
Group
Phenotype
Pre-term
Term
PRS Distribution for Preterm Birth
Weighted PRS Values
0.25
0.20
0.15
0.10
0.05
0.00
Pre-term
Term
Group
Phenotype
Pre-term
Term
PRS Distribution for Schizophrenia
Weighted PRS Values
0.2
0.1
0.0
Pre-term
Term
Group
Phenotype
Pre-term
Term

C)

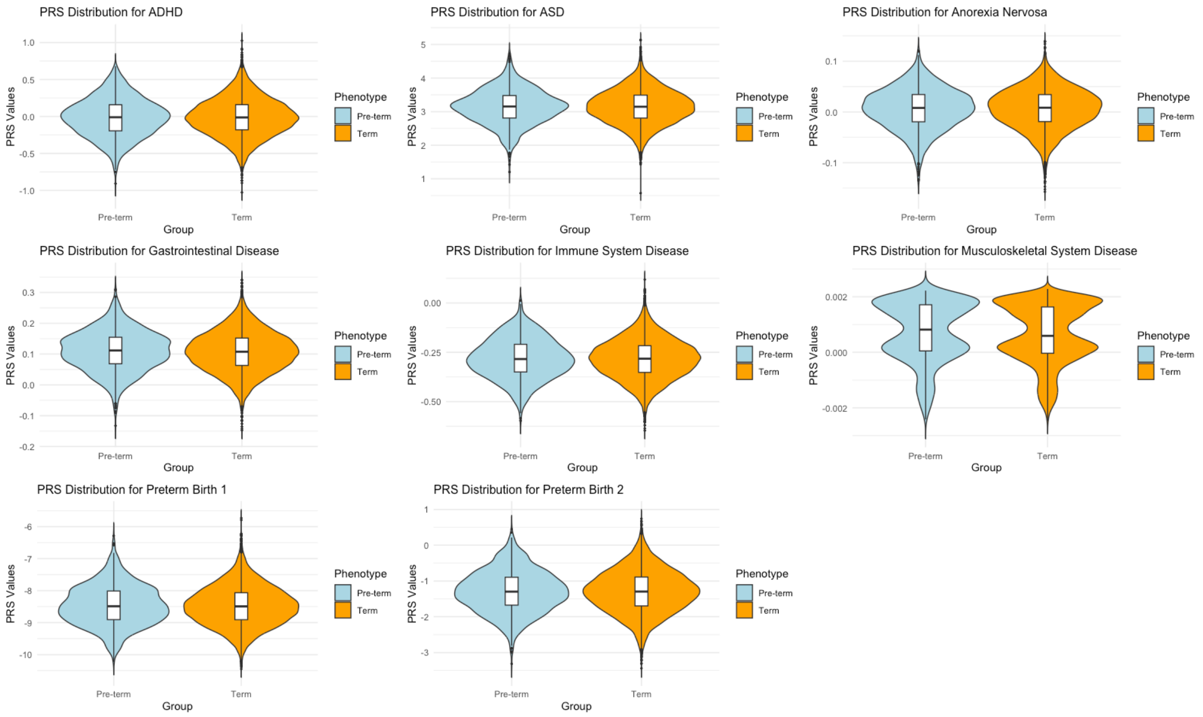

Figure S13. Violin plots displaying the distribution of PRS for different phenotypes in preterm (blue) and term (orange) groups of AMR (A), AFR (B) and EUR (C) ancestry. The distribution of PRS values is depicted using violin plots, with the width indicating the density of scores at different levels. Boxplots overlaid on each violin plot provide a summary of the PRS distribution, showing the median, quartiles, and outliers.

Supplemental Tables

Trait	AFR	AMR	EUR
**Preterm Birth**	AFR (Rappoport et al., 2018) https://doi.org/10.1038/s41598-017-18246-5	AMR (Rappoport et al., 2018) https://doi.org/10.1038/s41598-017-18246-5	EUR 1 (Liu et al., 2019) doi:10.1038/s41467-019-11881-8 & EUR 2 (Rappoport et al., 2018) https://doi.org/10.1038/s41598-017-18246-5
**Bipolar disorder**	AFR (O'Connell et al., 2024) https://doi.org/10.1101/2023.10.07.23296687	Trans-ancestry (O'Connell et al., 2024) https://doi.org/10.1101/2023.10.07.23296687	EUR (Mullins et al., 2021) DOI: 10.1038/s41588-021-00857-4
**Anxiety Disorders**	AFR (Friligkou et al., 2024) doi: 10.1038/s41588-024-01908-2	AMR (Friligkou et al., 2024) doi: 10.1038/s41588-024-01908-2	EUR (Friligkou et al., 2024) doi: 10.1038/s41588-024-01908-2
**SCZ**	AFR (Trubetskoy et al., 2022) https://doi.org/10.1038/s41586-022-04434-5	Trans-ancestry (Trubetskoy et al., 2022) https://doi.org/10.1038/s41586-022-04434-5	FinnGen (Kurki et al., 2023)
**Epilepsy**	Trans-ancestry (ILAE, 2023) https://doi.org/10.1038/s41588-023-01485-w	Trans-ancestry (ILAE, 2023) https://doi.org/10.1038/s41588-023-01485-w	Trans-ancestry (ILAE, 2014) DOI: 10.1016/S1474-4422(14)70171-1
**ADHD**	NA	NA	EUR (Demontis et al., 2023) https://doi.org/10.1038/s41588-022-01285-8
**ASD**	NA	NA	EUR (Grove et al., 2019) https://doi.org/10.1038/s41588-019-0344-8
**Anorexia Nervosa**	NA	NA	EUR (Watson et al., 2019) DOI: 10.1038/s41588-019-0439-2
**Immune System Disease**	NA	NA	EUR (Donertas et al., 2021) DOI: 10.1038/s43587-021-00051-5
**Musculoskeletal System Disease**	NA	NA	EUR (Donertas et al., 2021) DOI: 10.1038/s43587-021-00051-6
**Gastrointestinal Disease**	NA	NA	EUR (Donertas et al., 2021) DOI: 10.1038/s43587-021-00051-7

Table S1. Dataset Details for Each Trait and Ancestry.

Phenotype1	Phenotype2	rg	se	z	p
Panic Disorder	Meta	-0.789	0.642	-1.229	0.219
Gastrointestinal Disease	Meta	-0.053	0.081	-0.660	0.509
Panic Disorder	EUR	0.845	0.788	1.073	0.284
Preterm Birth (external)	Meta	-0.099	0.277	-0.358	0.720
Schizophrenia (EUR)	EUR	-0.083	0.036	-2.323	0.020
Anorexia Nervosa	Meta	-0.132	0.092	-1.432	0.152
Immune System Disease	EUR	0.081	0.054	1.506	0.132
Anorexia Nervosa	EUR	0.080	0.071	1.117	0.264
Immune System Disease	Meta	-0.066	0.086	-0.770	0.441
ASD	EUR	-0.117	0.072	-1.615	0.106
Musculoskeletal System Disease	EUR	0.070	0.049	1.441	0.150
Bipolar Disorder	EUR	-0.082	0.044	-1.889	0.059
Gastrointestinal Disease	EUR	0.004	0.052	0.073	0.942
Schizophrenia (all)	EUR	-0.062	0.031	-1.995	0.046
Musculoskeletal System Disease	Meta	-0.052	0.074	-0.701	0.483
Preterm Birth (external)	EUR	0.105	0.174	0.602	0.548
Schizophrenia (all)	Meta	0.090	0.050	1.805	0.071
Bipolar Disorder	Meta	0.117	0.071	1.650	0.099
ADHD	Meta	0.057	0.070	0.808	0.419
Epilepsy	EUR	0.009	0.086	0.106	0.916
ASD	Meta	0.204	0.115	1.770	0.077
Schizophrenia (EUR)	Meta	0.135	0.053	2.537	0.011
ADHD	EUR	0.025	0.043	0.584	0.559
Epilepsy	Meta	0.031	0.124	0.251	0.802

Table S2. LDSC analysis results in the full sample (preterm full) and restricted to individuals of European (EUR) ancestry.

Phenotype	Model	Val_obs	SE	Z	P(Z)
Preterm Birth (SPARK)	h1^2	0.296	0.122	2.428	0.015
ADHD	h2^2	0.044	0.005	8.064	0.000
ADHD	pge	-0.068	0.162	6.595	0.000
Preterm Birth (SPARK)	h1^2	0.282	0.131	2.154	0.031
Panic Disorder	h2^2	0.032	0.030	1.076	0.282
Panic Disorder	pge	0.556	0.643	0.691	0.490
Preterm Birth (SPARK)	h1^2	0.279	0.109	2.560	0.010
ASD	h2^2	0.140	0.039	3.625	0.000
ASD	pge	-0.146	0.232	4.935	0.000
Preterm Birth (SPARK)	h1^2	0.300	0.125	2.405	0.016
Bipolar Disorder	h2^2	0.045	0.005	8.557	0.000
Bipolar Disorder	pge	-0.130	0.150	7.546	0.000
Preterm Birth (SPARK)	h1^2	0.303	0.122	2.482	0.013
Anorexia Nervosa	h2^2	0.105	0.016	6.579	0.000
Anorexia Nervosa	pge	0.066	0.168	5.561	0.000
Preterm Birth (SPARK)	h1^2	0.326	0.135	2.421	0.015
Epilepsy	h2^2	0.080	0.014	5.567	0.000
Epilepsy	pge	0.190	0.228	3.552	0.000
Preterm Birth (SPARK)	h1^2	0.304	0.123	2.476	0.013
Gastrointestinal Disease	h2^2	0.013	0.002	5.231	0.000
Gastrointestinal Disease	pge	0.162	0.236	3.555	0.000
Preterm Birth (SPARK)	h1^2	0.296	0.111	2.675	0.007
Immune System Disease	h2^2	0.027	0.008	3.244	0.001
Immune System Disease	pge	0.069	0.136	6.866	0.000
Preterm Birth (SPARK)	h1^2	0.282	0.131	2.154	0.031
Musculoskeletal System Disease	h2^2	0.001	0.001	0.920	0.358
Musculoskeletal System Disease	pge	0.618	0.724	0.527	0.598
Preterm Birth (SPARK)	h1^2	0.301	0.124	2.425	0.015
Preterm Birth (external)	h2^2	0.008	0.009	0.819	0.413
Preterm Birth (external)	pge	0.760	0.677	0.355	0.723
Preterm Birth (SPARK)	h1^2	0.299	0.126	2.385	0.017
Schizophrenia (EUR)	h2^2	0.325	0.044	7.408	0.000
Schizophrenia (EUR)	pge	0.061	0.127	7.373	0.000
Preterm Birth (SPARK)	h1^2	0.299	0.126	2.384	0.017
Schizophrenia (all)	h2^2	0.245	0.024	10.006	0.000
Schizophrenia (all)	pge	0.055	0.127	7.465	0.000

Table S3. Popcorn results of genetic correlations between preterm birth and additional phenotypes in individuals of Admixed American (AMR) ancestry.

Phenotype	Model	Val_obs	SE	Z	P(Z)
Preterm Birth (SPARK)	h1^2	0.096	0.250	0.384	0.701
ADHD	h2^2	0.054	0.010	5.247	0.000
ADHD	pge	-0.579	0.947	1.667	0.095
Preterm Birth (SPARK)	h1^2	0.062	0.254	0.243	0.808
Panic Disorder	h2^2	0.088	0.056	1.555	0.120
Panic Disorder	pge	-1.798	5.158	0.543	0.587
Preterm Birth (SPARK)	h1^2	0.110	0.218	0.503	0.615
ASD	h2^2	0.120	0.027	4.463	0.000
ASD	pge	-0.348	0.716	1.883	0.060
Preterm Birth (SPARK)	h1^2	0.037	0.243	0.152	0.880
Bipolar Disorder	h2^2	0.073	0.010	6.984	0.000
Bipolar Disorder	pge	-0.031	0.721	1.431	0.152
Preterm Birth (SPARK)	h1^2	0.096	0.227	0.422	0.673
Anorexia Nervosa	h2^2	0.170	0.026	6.552	0.000
Anorexia Nervosa	pge	0.286	0.657	1.086	0.277
Preterm Birth (SPARK)	h1^2	0.167	0.336	0.496	0.620
Epilepsy	h2^2	0.083	0.026	3.213	0.001
Epilepsy	pge	-0.316	0.809	1.627	0.104
Preterm Birth (SPARK)	h1^2	0.101	0.240	0.420	0.674
Gastrointestinal Disease	h2^2	0.022	0.005	4.467	0.000
Gastrointestinal Disease	pge	-0.320	2.211	0.597	0.550
Preterm Birth (SPARK)	h1^2	0.083	0.229	0.364	0.716
Immune System Disease	h2^2	0.056	0.013	4.382	0.000
Immune System Disease	pge	0.689	1.217	0.255	0.799
Preterm Birth (SPARK)	h1^2	0.062	0.254	0.243	0.808
Musculoskeletal System Disease	h2^2	0.002	0.002	1.484	0.138
Musculoskeletal System Disease	pge	-1.358	4.026	0.586	0.558
Preterm Birth (SPARK)	h1^2	0.044	0.245	0.179	0.858
Preterm Birth (external)	h2^2	0.011	0.015	0.720	0.472
Preterm Birth (external)	pge	5.252	28.591	-0.149	0.882
Preterm Birth (SPARK)	h1^2	0.036	0.242	0.150	0.881
Schizophrenia (EUR)	h2^2	0.601	0.109	5.491	0.000
Schizophrenia (EUR)	pge	-0.739	5.140	0.338	0.735
Preterm Birth (SPARK)	h1^2	0.036	0.242	0.149	0.881
Schizophrenia (all)	h2^2	0.416	0.061	6.870	0.000
Schizophrenia (all)	pge	-0.716	5.290	0.324	0.746

Table S4. Popcorn results of genetic correlations between preterm birth and additional phenotypes in individuals of African/African American (AFR) ancestry.]

Phenotype	Model	Val_obs	SE	Z	P(Z)
Preterm Birth (SPARK)	h1^2	0.002	0.018	0.129	0.897
ADHD	h2^2	0.048	0.007	6.751	0.000
ADHD	pge	0.039	0.692	1.388	0.165
Preterm Birth (SPARK)	h1^2	0.011	0.019	0.565	0.572
Panic Disorder	h2^2	0.006	0.039	0.158	0.874
Panic Disorder	pge	-4.991	31.495	0.190	0.849
Preterm Birth (SPARK)	h1^2	0.005	0.019	0.266	0.791
ASD	h2^2	0.178	0.055	3.217	0.001
ASD	pge	0.474	1.192	0.441	0.659
Preterm Birth (SPARK)	h1^2	0.012	0.018	0.661	0.509
Bipolar Disorder	h2^2	0.059	0.008	7.337	0.000
Bipolar Disorder	pge	-0.167	0.432	2.702	0.007
Preterm Birth (SPARK)	h1^2	0.006	0.019	0.330	0.742
Anorexia Nervosa	h2^2	0.140	0.023	6.061	0.000
Anorexia Nervosa	pge	-0.070	0.629	1.702	0.089
Preterm Birth (SPARK)	h1^2	-0.006	0.024	-0.254	0.800
Epilepsy	h2^2	0.076	0.019	4.066	0.000
Epilepsy	pge	NaN	NaN	NaN	NaN
Preterm Birth (SPARK)	h1^2	0.012	0.019	0.646	0.518
Gastrointestinal Disease	h2^2	0.020	0.004	5.414	0.000
Gastrointestinal Disease	pge	-0.146	0.512	2.236	0.025
Preterm Birth (SPARK)	h1^2	0.012	0.019	0.618	0.536
Immune System Disease	h2^2	0.044	0.012	3.580	0.000
Immune System Disease	pge	0.160	0.660	1.273	0.203
Preterm Birth (SPARK)	h1^2	0.011	0.019	0.565	0.572
Musculoskeletal System Disease	h2^2	0.000	0.001	0.039	0.969
Musculoskeletal System Disease	pge	-9.922	104.429	0.105	0.917
Preterm Birth (SPARK)	h1^2	0.012	0.018	0.667	0.505
Preterm Birth (external)	h2^2	0.009	0.012	0.759	0.448
Preterm Birth (external)	pge	-1.192	3.635	0.603	0.546
Preterm Birth (SPARK)	h1^2	0.012	0.018	0.661	0.509
Schizophrenia (EUR)	h2^2	0.488	0.077	6.322	0.000
Schizophrenia (EUR)	pge	-0.401	0.451	3.109	0.002
Preterm Birth (SPARK)	h1^2	0.012	0.018	0.661	0.509
Schizophrenia (all)	h2^2	0.348	0.042	8.338	0.000
Schizophrenia (all)	pge	-0.226	0.375	3.270	0.001

Table S5. Popcorn results of heritability and genetic correlations between preterm birth and additional phenotypes in the entire sample (based on meta-analysis of ancestry-specific GWASs).

Category	Condition	OR [95%CI]	P-val	Q-val	Adj OR [95%CI]	Adj P-val	Adj Q-val
**Attention or behavioral conditions**	ADHD (Attention Deficit-Hyperactivity Disorder) or ADD	0.98 [0.93, 1.02]	0.344	0.905	0.98 [0.93, 1.02]	0.340	0.904
	Conduct Disorder	1.05 [0.90, 1.21]	0.550	0.905	1.04 [0.90, 1.21]	0.557	0.912
	Intermittent Explosive Disorder	0.96 [0.84, 1.10]	0.574	0.905	0.95 [0.83, 1.09]	0.472	0.912
	Oppositional Defiant Disorder	1.03 [0.96, 1.12]	0.415	0.905	1.03 [0.96, 1.12]	0.415	0.912
	Tourette Syndrome or Tic Disorder	0.98 [0.87, 1.10]	0.732	0.930	0.97 [0.86, 1.1]	0.670	0.912
**Birth defects**	Clubbed foot	1.29 [0.85, 1.96]	0.237	0.896	1.28 [0.84, 1.96]	0.252	0.904
	Missing or malformed bones	1.08 [0.78, 1.50]	0.641	0.909	1.08 [0.77, 1.5]	0.658	0.912
	Extra fingers and/or extra toes	1.14 [0.54, 2.39]	0.732	0.930	1.14 [0.54, 2.4]	0.736	0.944
	Spine deformity	1.49 [1.00, 2.22]	0.050	0.566	1.48 [0.99, 2.22]	0.056	0.632
	Cleft lip	0.82 [0.63, 1.08]	0.159	0.814	0.82 [0.63, 1.08]	0.159	0.718
	Cleft palate	0.68 [0.44, 1.06]	0.092	0.695	0.68 [0.44, 1.06]	0.093	0.699
	Brain malformation/abnormality (shown on MRI)	1.19 [0.85, 1.66]	0.310	0.905	1.19 [0.85, 1.66]	0.315	0.904
	Spina bifida/myelomeningocele (baby born with open spine, spinal cord outside of the body)	1.11 [0.63, 1.95]	0.724	0.930	1.11 [0.62, 1.96]	0.732	0.944
	Esophageal atresia (no connection between the esophagus and stomach)	0.69 [0.67, 0.70]	0.000	0.000	0.67 [0.64, 0.7]	0.000	0.000
	Hirschsprung disease	1.00 [0.98, 1.02]	1.000	1.000	1.00 [0.98, 1.02]	1.000	1.000
	Intestinal malrotation	0.94 [0.53, 1.67]	0.839	0.969	0.94 [0.54, 1.62]	0.810	0.950
	Pyloric stenosis (blockage from stomach to small intestine)	1.00 [0.98, 1.02]	1.000	1.000	1.00 [0.98, 1.02]	1.000	1.000
	Congenital diaphragmatic hernia	1.00 [0.98, 1.02]	1.000	1.000	1.00 [0.98, 1.02]	1.000	1.000
	Congenital heart disease/defect	0.94 [0.75, 1.19]	0.620	0.905	0.94 [0.75, 1.19]	0.617	0.912
	Lung malformation	0.93 [0.75, 1.17]	0.545	0.905	0.93 [0.73, 1.18]	0.544	0.912
	Hypospadias (in boys, urinary opening is in the wrong place)	1.08 [0.76, 1.55]	0.668	0.926	1.08 [0.76, 1.55]	0.659	0.912
	Kidney malformation (for example horseshoe kidney)	0.88 [0.53, 1.46]	0.614	0.905	0.87 [0.52, 1.45]	0.593	0.912
	Missing kidney	2.36 [2.24, 2.48]	0.000	0.000	2.38 [2.25, 2.52]	0.000	0.000
	Missing uterus	0.41 [0.39, 0.42]	0.000	0.000	0.42 [0.34, 0.51]	0.000	0.000
**Birth/pregnancy complications**	Fetal Alcohol Syndrome, alcohol or drug exposure in mother's pregnancy	1.15 [0.94, 1.40]	0.168	0.814	1.15 [0.94, 1.4]	0.169	0.718
	Bleed into the brain	1.12 [0.87, 1.44]	0.393	0.905	1.12 [0.87, 1.44]	0.400	0.912

	Insufficient oxygen at birth with NICU stay	0.96 [0.86, 1.06]	0.421	0.905	0.96 [0.86, 1.06]	0.421	0.912
	Serious prenatal infection (for example, German measles)	1.34 [0.92, 1.95]	0.123	0.767	1.34 [0.92, 1.94]	0.123	0.718
	Premature birth (delivery before 37 weeks)	1.01 [0.94, 1.09]	0.808	0.963	1.01 [0.94, 1.09]	0.805	0.950
**Developmental conditions**	Cognitive delays or impairment due to another medical condition or exposure (For example, brain injury, stroke, lead poisoning, FAS, HIV, radiation, hydrocephalus, brain tumor, drug effects, etc.)	1.03 [0.93, 1.13]	0.609	0.905	1.02 [0.93, 1.13]	0.629	0.912
	Intellectual disability, cognitive impairment, global developmental delay, or borderline intellectual functioning	1.01 [0.96, 1.07]	0.616	0.905	1.01 [0.96, 1.07]	0.607	0.912
	Language delay or language disorder	1.00 [0.95, 1.04]	0.872	0.969	1.00 [0.95, 1.05]	0.883	0.969
	Learning disability (LD, learning disorder, including reading, written expression, math, or NVLD (Nonverbal learning disability))	0.96 [0.91, 1.01]	0.129	0.767	0.96 [0.91, 1.01]	0.123	0.718
	Motor delay (e.g., delay in walking) or developmental coordination disorder	0.99 [0.94, 1.05]	0.784	0.959	0.99 [0.94, 1.05]	0.783	0.950
	Mutism	1.18 [0.95, 1.47]	0.135	0.767	1.18 [0.95, 1.47]	0.140	0.718
	Social (Pragmatic) Communication Disorder	1.03 [0.97, 1.09]	0.393	0.905	1.03 [0.97, 1.1]	0.346	0.904
	Speech articulation problems	0.98 [0.93, 1.04]	0.554	0.905	0.98 [0.93, 1.04]	0.543	0.912
	Cognitive delays or impairment due to another medical condition or exposure (For example, brain injury, stroke, lead poisoning, FAS, HIV, radiation, hydrocephalus, brain tumor, drug effects, etc.)	1.03 [0.93, 1.13]	0.609	0.905	1.02 [0.93, 1.13]	0.629	0.912
**Growth related conditions**	Difficulty gaining weight	1.04 [0.92, 1.18]	0.494	0.905	1.04 [0.92, 1.18]	0.496	0.912

	Large head size (macrocephaly)	1.05 [0.93, 1.20]	0.416	0.905	1.06 [0.94, 1.21]	0.339	0.904
	Small head size (microcephaly)	1.11 [0.92, 1.34]	0.284	0.905	1.11 [0.92, 1.33]	0.291	0.904
	Obesity	0.99 [0.87, 1.12]	0.889	0.969	0.98 [0.86, 1.11]	0.704	0.938
	Short stature	1.08 [0.94, 1.25]	0.286	0.905	1.08 [0.94, 1.25]	0.288	0.904
**Mood/psychiatric conditions**	Alcohol or Substance Use	0.87 [0.68, 1.10]	0.236	0.896	0.81 [0.62, 1.07]	0.138	0.718
	Anxiety disorder, such as panic, phobia, agoraphobia, or generalized anxiety disorder (GAD) except for social anxiety	0.99 [0.94, 1.04]	0.625	0.905	0.98 [0.93, 1.04]	0.480	0.912
	Bipolar (Manic-Depressive) Disorder	1.03 [0.92, 1.16]	0.574	0.905	1.02 [0.90, 1.15]	0.768	0.950
	Depression or dysthymia	1.04 [0.98, 1.11]	0.192	0.818	1.04 [0.97, 1.12]	0.307	0.904
	Disruptive Mood Dysregulation Disorder	1.12 [0.99, 1.27]	0.069	0.675	1.12 [0.99, 1.27]	0.068	0.662
	Hoarding	1.02 [0.89, 1.17]	0.790	0.959	1.01 [0.88, 1.16]	0.846	0.959
	Obsessive-Compulsive Disorder	1.00 [0.94, 1.07]	0.902	0.969	1.00 [0.93, 1.07]	0.968	1.000
	Separation Anxiety	0.99 [0.91, 1.08]	0.871	0.969	0.99 [0.91, 1.08]	0.850	0.959
	Social Anxiety Disorder/Social Phobia	0.98 [0.92, 1.04]	0.439	0.905	0.97 [0.90, 1.03]	0.319	0.904
	Personality Disorder	1.28 [1.05, 1.56]	0.015	0.248	1.28 [1.02, 1.61]	0.032	0.552
	Schizophrenia, Other Psychosis or Schizoaffective Disorder	1.04 [0.86, 1.25]	0.713	0.930	1.02 [0.84, 1.24]	0.808	0.950
**Neurological conditions**	Brain infection such as bacterial meningitis, encephalitis	0.72 [0.52, 1.00]	0.050	0.566	0.71 [0.51, 0.99]	0.046	0.622
	Lead poisoning	1.02 [0.70, 1.49]	0.907	0.969	1.01 [0.69, 1.48]	0.940	0.999
	Seizure disorder or epilepsy	0.97 [0.89, 1.06]	0.524	0.905	0.97 [0.89, 1.06]	0.508	0.912
	Traumatic brain injury (hospitalized)	0.89 [0.69, 1.15]	0.377	0.905	0.88 [0.68, 1.14]	0.343	0.904
**Sleep/feeding/eating/ toileting related conditions**	Problems with eating foods - not diagnosed by a professional	1.01 [0.96, 1.06]	0.594	0.905	1.01 [0.96, 1.06]	0.664	0.912
	Eating Disorder	0.91 [0.70, 1.17]	0.450	0.905	0.88 [0.66, 1.18]	0.397	0.912
	Encopresis (has bowel accidents beyond age expected)	1.08 [0.99, 1.17]	0.084	0.695	1.08 [0.99, 1.17]	0.083	0.699
	Enuresis (wets self beyond age expected)	1.00 [0.93, 1.09]	0.928	0.971	1.00 [0.92, 1.09]	0.927	0.999
	Feeding/eating problems	0.96 [0.90, 1.02]	0.193	0.818	0.95 [0.89, 1.02]	0.163	0.718
	Sleep Disorder or sleep problem diagnosed by a professional	1.02 [0.96, 1.07]	0.535	0.905	1.02 [0.96, 1.07]	0.579	0.912

	Sleep problems not diagnosed by a professional	1.00 [0.95, 1.05]	0.912	0.969	1.00 [0.95, 1.05]	0.860	0.959
**Vision/hearing conditions**	Blindness	0.88 [0.64, 1.21]	0.445	0.905	0.88 [0.64, 1.21]	0.443	0.912
	Cataract	0.93 [0.60, 1.43]	0.738	0.930	0.91 [0.58, 1.42]	0.671	0.912
	Deafness/hearing loss	1.06 [0.92, 1.23]	0.394	0.905	1.06 [0.92, 1.23]	0.431	0.912
	Strabismus	1.05 [0.93, 1.19]	0.460	0.905	1.05 [0.93, 1.19]	0.465	0.912

Table S6. Associations between preterm PRS in the SPARK data and reported medical conditions. Preterm PRS was derived using summary statistics from preterm birth GWAS in an external (population-based) sample to avoid overfitting, using BridgePRS. Adjusted estimates were additionally adjusted for child's year of birth and sex.

Phenotype	Est	2.5%	97.5%	Significant
ADHD	0.000	0.000	0.000	FALSE
ASD	0.000	-0.001	0.000	FALSE
Anorexia Nervosa	0.000	-0.001	0.000	FALSE
Gastrointestinal Disease	0.000	-0.001	0.001	FALSE
Immune System Disease	0.000	0.000	0.000	FALSE
Musculoskeletal System Disease	0.001	-0.001	0.003	FALSE
Preterm Birth 1	0.000	0.000	0.000	FALSE
Preterm Birth 2	0.000	-0.001	0.000	FALSE

Table S7. Association between preterm status (reported) in the SPARK sample and genetic liability to additional medical conditions in individuals of European (EUR) ancestry. PRS for all traits was calculated using BridgePRS.

Phenotype	Model	Prob	Est	2.5	97.5	Significant	Ancestry
Anxiety Disorder	Stage1	0.260	-0.001	-0.005	0.001	FALSE	AFR
Anxiety Disorder	Stage2	0.358	0.000	0.000	0.000	FALSE	AFR
Anxiety Disorder	Stage1+2	0.382	0.000	0.000	0.000	FALSE	AFR
Anxiety Disorder	Weighted	1.000	-0.001	-0.005	0.001	FALSE	AFR
Bipolar Disorder	Stage1	0.326	0.000	-0.006	0.004	FALSE	AFR
Bipolar Disorder	Stage2	0.345	0.000	0.000	0.000	FALSE	AFR
Bipolar Disorder	Stage1+2	0.329	0.000	-0.006	0.004	FALSE	AFR
Bipolar Disorder	Weighted	1.000	0.000	-0.006	0.004	FALSE	AFR
Epilepsy	Stage1	0.246	-0.001	-0.005	0.001	FALSE	AFR
Epilepsy	Stage2	0.538	0.000	0.000	0.000	FALSE	AFR
Epilepsy	Stage1+2	0.216	0.000	0.000	0.000	FALSE	AFR
Epilepsy	Weighted	1.000	-0.001	-0.005	0.001	FALSE	AFR
Preterm Birth (external)	Stage1	0.301	0.000	0.000	0.000	FALSE	AFR
Preterm Birth (external)	Stage2	0.352	0.000	0.000	0.000	FALSE	AFR
Preterm Birth (external)	Stage1+2	0.347	0.000	0.000	0.000	FALSE	AFR
Preterm Birth (external)	Weighted	1.000	0.000	0.000	0.000	FALSE	AFR
Schizophrenia	Stage1	0.018	-0.001	-0.006	0.002	TRUE	AFR
Schizophrenia	Stage2	0.787	0.004	-0.006	0.012	FALSE	AFR
Schizophrenia	Stage1+2	0.194	0.003	-0.005	0.010	FALSE	AFR
Schizophrenia	Weighted	1.000	0.004	-0.006	0.012	FALSE	AFR
Anxiety Disorder	Stage1	0.210	0.000	-0.002	0.001	FALSE	AMR
Anxiety Disorder	Stage2	0.450	0.000	-0.002	0.001	FALSE	AMR
Anxiety Disorder	Stage1+2	0.339	0.000	-0.002	0.000	FALSE	AMR
Anxiety Disorder	Weighted	1.000	0.000	-0.002	0.000	FALSE	AMR
Bipolar Disorder	Stage1	0.344	0.000	-0.003	0.001	FALSE	AMR
Bipolar Disorder	Stage2	0.423	0.000	-0.002	0.000	FALSE	AMR
Bipolar Disorder	Stage1+2	0.234	0.000	-0.003	0.001	FALSE	AMR
Bipolar Disorder	Weighted	1.000	0.000	-0.002	0.001	FALSE	AMR
Epilepsy	Stage1	0.227	0.000	-0.003	0.002	FALSE	AMR
Epilepsy	Stage2	0.546	0.000	-0.003	0.001	FALSE	AMR
Epilepsy	Stage1+2	0.228	0.000	0.000	0.000	FALSE	AMR
Epilepsy	Weighted	1.000	0.000	-0.003	0.001	FALSE	AMR
Preterm Birth (external)	Stage1	0.223	0.000	0.000	0.000	FALSE	AMR
Preterm Birth (external)	Stage2	0.502	0.000	-0.002	0.000	FALSE	AMR
Preterm Birth (external)	Stage1+2	0.274	0.000	-0.003	0.002	FALSE	AMR
Preterm Birth (external)	Weighted	1.000	0.000	-0.002	0.001	FALSE	AMR
Schizophrenia	Stage1	0.233	0.000	-0.002	0.001	FALSE	AMR
Schizophrenia	Stage2	0.486	0.000	-0.002	0.000	FALSE	AMR
Schizophrenia	Stage1+2	0.281	0.000	-0.002	0.000	FALSE	AMR
Schizophrenia	Weighted	1.000	0.000	-0.002	0.000	FALSE	AMR

Table S8. Association between preterm status (reported) in the SPARK sample and genetic liability to additional medical conditions in individuals of African / African American (AFR) and Admixed American (AMR) ancestry. PRS for all traits was calculated using BridgePRS.
